# Do We Know Enough About the Safety Profile of Silver Nanoparticles in Oncology? A Focus on Novel Methods and Approaches

**DOI:** 10.3390/ijms26115344

**Published:** 2025-06-02

**Authors:** Peter Takáč, Radka Michalková, Martina Čižmáriková, Zdenka Bedlovičová, Ľudmila Balážová, Štefánia Laca Megyesi, Zuzana Mačeková, Gabriela Takáčová, Almudena Moreno-Borrallo, Eduardo Ruiz-Hernandez, Luka Isakov, Peter Takáč

**Affiliations:** 1 Department of Pharmacology and Toxicology, University of Veterinary Medicine and Pharmacy in Košice, Komenského 73, 041 81 Košice, Slovakia; 2 Department of Pharmacology, Faculty of Medicine, Pavol Jozef Šafárik University, 040 01 Košice, Slovakia; 3 Department of Chemistry, Biochemistry and Biophysics, University of Veterinary Medicine and Pharmacy in Košice, Komenského 73, 041 81 Košice, Slovakia; 4 Department of Pharmaceutical Technology, Pharmacognosy and Botany, University of Veterinary Medicine and Pharmacy in Košice, 041 81 Košice, Slovakia; 5 Department of Pharmacy and Social Pharmacy, University of Veterinary Medicine and Pharmacy in Košice, 041 81 Košice, Slovakia; 6 Department of Dermatovenerology, Faculty of Medicine, Pavol Jozef Šafárik University and L. Pasteur University Hospital in Košice, 040 01 Košice, Slovakia; 7 School of Pharmacy and Pharmaceutical Sciences, Trinity College Dublin, D02 YY50 Dublin, Ireland; 8 BioSense Institute, University of Novi Sad, Dr. Zorana Đinđića 1, 21102 Novi Sad, Serbia; 9 Department of Physical Medicine, Balneology and Medical Rehabilitation, Faculty of Medicine, Pavol Jozef Šafárik University and L. Pasteur University Hospital in Košice, 040 01 Košice, Slovakia

**Keywords:** nano-oncology, silver, safety assessment, novel methods

## Abstract

Silver nanoparticles (AgNPs) have emerged as promising agents in cancer diagnostics and/or therapy, demonstrating a lot of possible pharmacological actions. However, understanding the pharmacokinetics and safety profiles of nanoparticles, which is crucial for their clinical application, still raises many questions. Studies indicate that AgNPs can accumulate in tumour tissues, improving drug delivery and specificity. However, their interaction with biological systems necessitates thorough safety evaluations. Classical methods for assessing AgNPs’ safety include cytotoxicity assays, genotoxicity tests, and histopathological examinations. However, novel techniques are emerging, such as advanced imaging and biomarker analysis, offering more precise toxicity assessments. Prediction models, including computational simulations and in silico analyses, are being developed to forecast AgNPs’ toxicity profiles. These models aim to reduce reliance on animal testing and expedite the evaluation process. To mitigate potential risks associated with nanoparticle-based therapies, strategies such as surface modification, controlled release systems, and targeted delivery are being explored. These methods aim to enhance therapeutic efficacy while minimizing adverse effects. The main aim of this review article is to describe AgNPs from the point of view of their pharmacokinetic/toxicokinetic profile in the light of modern knowledge. Special attention will be given to novel methods for assessing the safety and toxicity profiles of AgNPs, providing insights into their interactions with cancer therapies and their potential clinical applications.

## 1. Introduction

Nanoparticles have attracted considerable attention in recent decades due to their distinctive physiochemical properties, serving as therapeutic agents [1,2,3], catalysts [4,5], and enhancers of commercial materials [6,7,8]. The small size and unique chemistry of nanomaterials modify or enhance bulk material properties, resulting in new characteristics that are not achievable by macroscale materials.

Nano silver exemplifies a substance whose applications are undergoing significant transformation. Numerous studies have concentrated on the therapeutic targets of silver nanoparticles (AgNPs) [9,10,11]. In our previous review, we summarized the in vitro and in vivo anticancer mechanisms of AgNPs, as well as their potential in cancer diagnosis [12].

On the other hand, the possibility of safe clinical use of AgNPs has not been fully elucidated. Thus, more detailed research should be undertaken to evaluate the biocompatibility and potential cell toxicity of AgNPs. This may facilitate the development of more secure and biocompatible agents derived from AgNPs. Furthermore, an in vitro-to-in vivo extrapolation is necessary to substantiate the advancement of the next-generation risk assessment (NGRA) strategy for AgNPs [13].

Novel approaches and techniques such as computational simulations, multi-omics strategies, Organ-on-a-Chip (OoC) platforms, etc., are necessary to forecast AgNPs’ toxicity profiles. These models seek to decrease dependence on animal testing, accelerate the evaluation process, and, last but not least, improve therapeutic efficacy while reducing adverse effects.

The main aim of this article is to describe the safety profile of AgNPs and related information regarding their pharmacokinetic, toxicokinetic, and biosafety characteristics. Special attention will be given to novel methods for assessing AgNPs’ safety and toxicity profiles.

## 2. Synthesis of Silver Nanoparticles

Silver nanoparticle synthesis is usually divided into two main groups of approaches—top-down (including physical synthesis) and bottom-up methods (chemical and biological synthesis) (Table 1).

**Figure 1:**
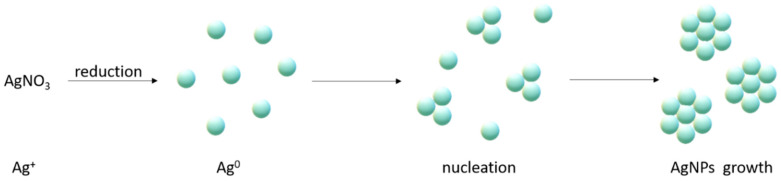
Mechanism of synthesis by Ag^+^ salt reduction.

The bottom-up methods are generally understood as constructive techniques, representing chemical and biological methods of silver nanoparticle (AgNP) synthesis. Chemical methods, in general, serve as an easy way for AgNP preparation in solution (an organic solvent or water is usually used). These methods are valuable due to their low cost and large-scale capacity of production. The synthesis is based on the reduction of a silver salt, serving as a precursor of Ag+ ions (mainly silver nitrate), by a reducing agent. NaBH_4_, H_2_, sodium ascorbate, and sodium citrate are usually used in the reduction process. Finally, the presence of a stabilizing agent, such as (PVP—poly(vinyl)pyrrolidone, PVA—poly(vinyl)alcohol, PEG—polyethylene glycol, or less commonly used surfactants like CTAB—cetyltrimethylammonium bromide and AOT—sodium bis(2-ethylhexyl) sulphosuccinate), is required to protect against agglomeration. The formation mechanism of silver NPs is induced by generating silver in its neutral form (Ag^0^) followed by nucleation (high activation energy is required) and growth (Figure 1). The advantage of the chemical approach for AgNP synthesis is good shape and size control through the ability to adjust the reaction conditions (pH, temperature, concentration) [14,15,16,17,18,19,20].

**Figure 2:**
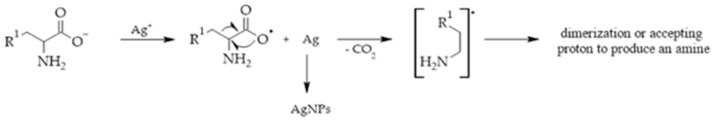
Proposed mechanism of AgNP synthesis by α-amino acids [21].

Variations of this type of reaction have been described. An interesting study on the synthesis of AgNPs by α-amino acids was published by Kumar et al. [21]. The authors reported that aromatic amino acids (phenylalanine, tryptophan, tyrosine) provided better yields, whereas cysteine, for example, provided no AgNP due to the complexation of the sulphur atom with Ag+ ions. The authors also proposed a mechanism of reaction, in which the α-amino acid reacts with a silver (1+) ion to form a free radical of amino acid, either dimerizing or accepting a proton, yielding an amine (Figure 2) [21].

The sonochemical approach is safe and fast, providing good control of reaction conditions [21,22,23,24,25]. The essential mechanism of nanoparticle synthesis, sonochemically, is based on the cavitation effect, which leads to the conversion of the solvent (mainly water) into free radicals (H·, OH·, HO_2_·) able to provide adequate chemical potential, reducing the metallic ion [25]. The sonochemical method is in contrast with the classical reduction reaction. Sonochemical synthesis does not require high temperatures, a long time for the reaction to take place, shape control, or a capping reagent. It is a good technique for preparing NPs around 15 nm with specified physical properties, including specific size and shape requirements [22].

Photochemical and microwave-assisted syntheses are based on the exposure of a metallic ion solution to ultraviolet or visible light and microwave irradiation, respectively. The processes are initialised by reducing a silver ion (1+) into its elemental form and then adding a stabilisation agent [26,27].

The electrochemical procedure of AgNP synthesis is based on dissolving the metallic anode in an aprotic solvent. An Ag/AgCl reference electrode is usually used, with a silver sheet as an anode, and a platinum sheet as a cathode. The solvent is required to be oxygen-free to prevent the oxidation of small generating particles [28].

A biological approach is one of the green synthesis approaches for AgNP fabrication. The advantage of this approach is that it is environmentally friendly due to preventing the use of toxic and harmful chemical reagents and lowering energy consumption. The reduction of silver ions is performed using a biological system. The biological system itself serves as a reducing and capping agent. Useful biological sources are mainly plant extracts, but also microorganisms and biomolecules such as saccharides, polysaccharides, proteins, and amino acids [29,30,31,32,33,34].

**Figure 3:**
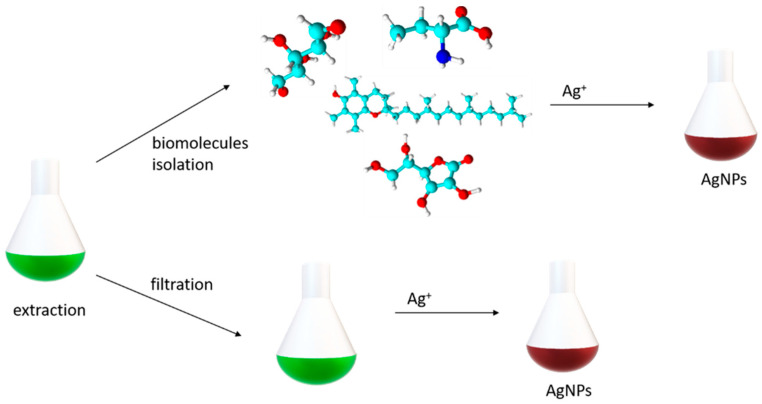
Schematic representation of biological silver nanoparticle synthesis using extraction (in vitro).

The synthesis of silver NPs using the biological approach is also based on the reduction of silver ions into elemental silver, resulting in growth and stabilisation (Figure 3) [35]. As we mentioned in Table 1, biological methods can be divided into in vivo and in vitro methods, hence intracellular and extracellular methods. The intracellular, in vivo method is typically used for Ag nanoparticle synthesis by living organisms. The first step of in vivo synthesis is culturing the organism, then the living cells react with the Ag ion precursor. The final step is to separate and purify the NPs [34,36].

It is a well-known fact that some species of microorganisms and plants are capable of absorbing and accumulating metallic ions from the environment in which they are grown. These features make living organisms effective “biological factories”, with the ability to reduce environmental pollution. The very first report on this problem was studied by Gardea-Torresday’s group, which used a living plant, alfalfa (Medicago salvia), to prepare AgNPs in vivo. The plant root absorbed silver ions from the nutrient medium under controlled conditions. The Ag+ ions were absorbed by the whole plant to produce silver NPs [37]. Other authors also successfully synthesised AgNPs using the living plants *Festuca rubra, Medicago sativa*, and *Brassica juncea* [38]. The fast bio-reduction was performed within 24 h of exposition to silver nitrate solution. TEM analyses determined the in vivo formation of AgNPs in the roots, stems, and leaves of the plants with similar distributions, but various sizes and shapes. The presence of reducing saccharides and antioxidant compounds, such as flavonoids and phenolics, was proposed to be responsible for silver ion reduction in AgNPs biosynthesis.

Another successful biosynthesis of AgNPs was conducted using microorganisms. Microorganisms have the ability to utilise their enzymatic processes for nanoparticle synthesis. There is an important need to note that not all organisms have the capacity to synthesise these nanoparticles, due to biochemical enzymatic activities and metabolic processes, so a thoughtful choice of biological system is required [38]. The culturing conditions, such as pH, nutrients, temperature, buffer, inoculum size, time, and light, must be optimised and are very essential for biosynthesis. The substrate’s participation from the initialisation of growth increases the activity of enzymes [30,39,40,41,42]. The very first microorganism used for AgNP synthesis was the *Pseudomonas stutzeri* AG259 strain used by Klaus and her colleagues in 1999 [43]. Since that time, some more reports have focused on the biosynthesis of AgNPs by microorganisms, namely *Bacillus methylotrophicus* [44], *Lactobacillus strain* [45,46], and *E. coli* [47].

The size and monodispersity control of synthesised AgNPs is important. Each microbe genus is capable of creating different shapes and sizes of nanoparticles [32]. Living human cancer cells (human cervical cancer cell line—HeLa, human embryonic kidney cells—HEK293T, and human liver carcinoma cell line—HepG2) were also successfully used for Au and AgNP fabrication inside the cell. Finally, the presence of NPs inside the cell led to cell lysis [48].

The in vitro approach utilises biomolecules that are isolated from natural sources. The extraction and purification of biomolecules is necessary, so it tends to be a time-consuming experiment. The use of simple, biologically occurring molecules, such as saccharides (glucose), polysaccharides (chitosan, dextrin, cellulose), and amino acids [49,50,51,52,53,54,55,56,57], leads to the simplification of nanoparticle synthesis (Figure 3).

An uncountable number of studies can be found in the field of plant-mediated synthesis of silver nanoparticles. A wide variety of plant species and specific parts of plants have been used [58,59,60]. Generally, silver nitrate solution is the precursor of silver ions and the extract obtained from the plant is used for Ag+ ion reduction and stabilisation of the obtained nanoparticles [31,61]. Some papers deal with the synthesis of AgNPs by essential oils as an alternative to plant extracts [62,63,64]. Interestingly, microorganism (fungi, bacteria, yeasts, actinomycetes) extracts have also been used to synthesise nanoparticles with characteristic morphology. Analogically, the extracts obtained from microorganisms may serve as reducing and stabilising agents due to biomolecules (amino acids, enzymes, polysaccharides or vitamins). The extraction is conducted by washing the biomass and dissolving the cells in water or a buffer [65], or by using the medium in which the biomass was grown [66]. One of the methods for silver NP synthesis uses a supernatant of *Bacillus subtilis* and microwave irradiation to avoid aggregation in water solution [67]. The field of extracellular AgNP synthesis using microorganisms is broadly studied and a lot of reports have been released [68]. Mushrooms, especially edible ones, are known as a source of proteins, (poly)saccharides, vitamins, and polyphenols, so they can also be used as producers of Ag nanoparticles. Various mushrooms were tested for their ability to synthesise nanoparticles [69,70,71,72,73].

The top-down approach is understood as a destructive technique—from bulk material, the smaller molecules are prepared. Physical methods depend on energy sources to reduce particle size, for example mechanical energy (ball milling), light energy (laser ablation), or thermal energy (thermal decomposition) [74]. The disadvantages of physical methods are high energy consumption and low reaction yield. On the other hand, the main advantage is the rate of the reactions and the avoidance of toxic chemicals [75]. A very interesting and relatively new method for silver NP preparation is ball milling. This technique belongs to the category of mechanical methods for AgNP synthesis and uses milling balls, which are placed into a container. The metal reagent (for example, silver nitrate in the solid state) and a specific mass are rotated at a defined rate. The time and rate of milling, ratio of salt, and mass play an essential role in the final morphology of the nanoparticles [74,76]. Laser ablation is based on instantly heating the bulk material by pulsing laser light in a solvent (an organic solvent such as water) to form a plasma plume. The plasma plume is then subjected to nucleation and growth by the NPs [74,77]. Thermal decomposition is an endothermic process, when bulk material is decomposed. The specific temperature at which the element is decomposed is called the temperature of decomposition [19]. Generally, the physical (top-down) methods are relatively fast and nanoparticles are usually pure and of uniform size, but the instrumentation and energy consumption are economically demanding.

## 3. Characterisation Techniques

The characterisation of nanoparticles, in general, is very important for the evaluation of their physical and physicochemical properties, which determine their behaviour, biological distribution, and safety. A broad spectrum of methods is used for AgNP characteristics evaluation. The most common techniques include infrared (FTIR) spectroscopy, ultraviolet and visible (UV/Vis) spectroscopy, X-ray diffraction (XRD), X-ray photoelectron spectroscopy (XPS), dynamic light scattering (DLS), zeta potential analysis, scanning electron microscopy (SEM), and transmission (TEM) electron microscopy.

**Figure 4:**
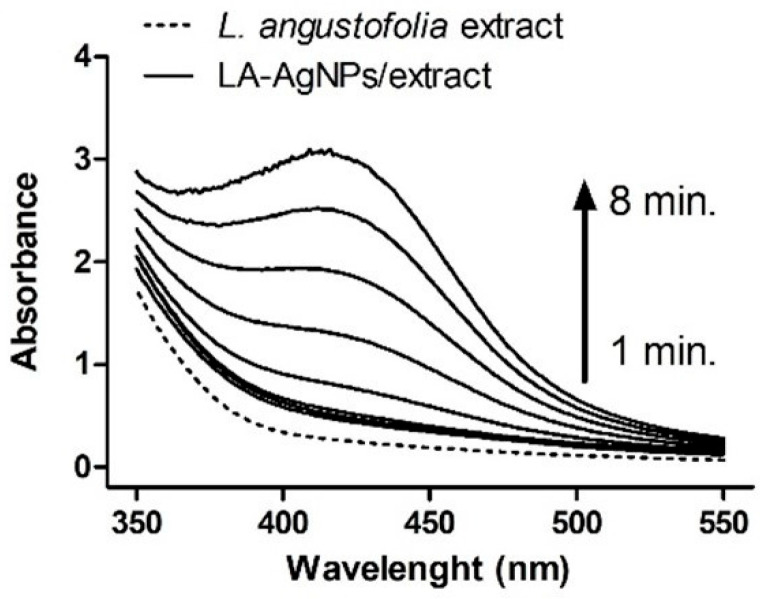
Time-dependent UV-Vis spectra of AgNP synthesis with detection of SPR bands using *Lavandula angustifolia* plant extract and 5 mM AgNO_3_ as precursor [61].

The UV/Vis method is widely used primarily for the early detection of nanoparticle synthesis. This technique is available, simple, and sensitive for measuring and monitoring the reaction process in nanoparticle preparation [78]. The simplicity of monitoring the reaction process is based on surface plasmon resonance (SPR) band detection during the synthesis of silver nanoparticles. SPR refers to the coherent oscillation of the electrons present on the surface of particles that are excited by electromagnetic radiation [79,80]. The position of the SPR band depends on the nanoparticle size, dielectric medium, and chemical environment, so these bands can serve as stability detection for prepared AgNPs [75,81]. An example of the UV/Vis spectra representing SPR bands of silver NPs synthesized by Lavandula angustifolia water extract and aqueous silver nitrate as the precursor is presented in Figure 4 [61]. As we can see, in the fourth minute after mixing the lavender extract and silver nitrate solution, the absorbance in the region around 412 nm started to increase, and after 8 min, it reached its maximum [61].

**Figure 5:**
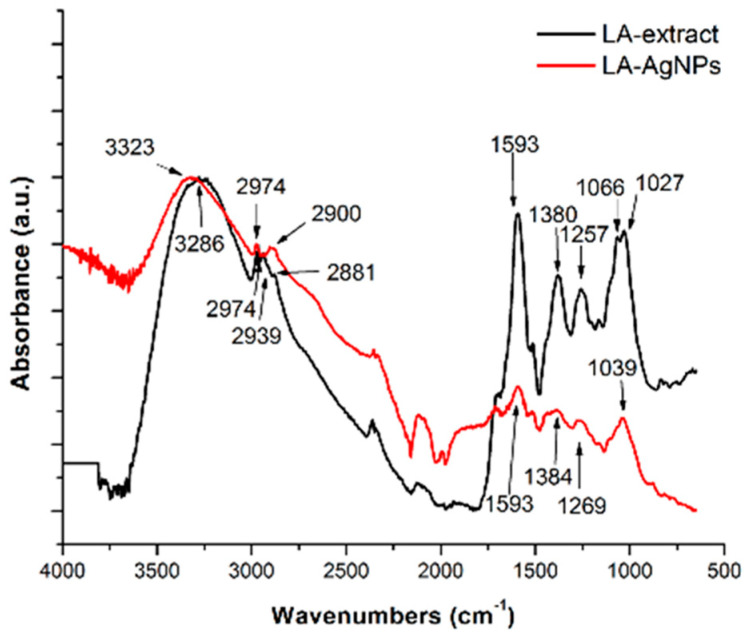
Attenuated total reflectance ATR-FTIR absorption spectra of *Lavandula angustifolia* plant extract and AgNPs prepared from 5 mM AgNO_3_ as precursor [61].

FTIR (Fourier transform infrared spectroscopy) is a valuable method that is used for the identification of functional groups responsible for the reduction of silver nitrate ions during AgNP preparation, due its ability to detect small changes in molecule functionalities, e.g., interactions between biomolecules and nanomaterial [82,83]. An additional method to FTIR is attenuated total reflection ATR-FTIR spectroscopy, which may serve as a good technique for studying the surface characteristics of nanomaterials. The disadvantage of this method is that it does not have good sensitivity at the nanoscale range due to its penetration depth and it also has the same order of magnitude as the infrared wavelength [83,84]. An example of an ATR-FTIR spectrum from the same study as the UV/Vis spectrum is shown in Figure 5 [61].

ATR-FTIR analysis of the dried L. angustifolia extract and AgNPs showed that the extract and Ag nanoparticles had some slight shifts and changed band intensities. The main identified functional groups were -OH, C-H, C=C, and -C-O-, as are shown in Figure 5. Silver nitrate was not detected in this spectrum [61].

X-ray diffraction (XRD) is based on the reflection of X-ray light on studied material, leading to the formation of diffraction peaks reflecting structural characteristics. This analytical method is usually used for the identification of molecular and crystal structures and also particle size in various systems including polymers, catalysts, biomolecules, nanostructures, and impurities [85,86,87,88]. This technique is applicable in the synthesis of silver NPs because silver and silver nitrate are easily recognisable and we can see the progress of the synthesis [89].

X-ray photoelectron spectroscopy (XPS) is used for obtaining qualitative and quantitative surface analysis data, providing chemical state information from the elements (except hydrogen and helium) present in the sample. The X-rays bombard the surface of the studied material, including nanoparticles, which leads to the emission of electrons. The kinetic energy of emitted electrons is measured. The amount of leaked electrons from the nanoparticle’s surface provides the XPS spectra [75,90,91].

Zeta potential (ZP) provides information about nanoparticle-bioconjugate charge and stability by measuring electrokinetic potential [92]. The AgNPs prepared from *L. angustifolia*, as presented in our previous work, exhibited a ZP of −15.8 mV and pH of 3.31. The negative value of ZP was detected, indicating the relatively high stability of the nanosuspension, because values lower than 30 mV predict stable nanoparticles [61,62].

The dynamic light scattering (DLS) method plays its role in measuring the size distribution of small particles in solution or suspension [86,88]. The laser light is passed through a sample and interacts with nanoparticles (or particles). This method is used for measuring scattered light intensity as a function of time, and the average hydrodynamic size of nanoparticles dispersed in a liquid medium is determined [75,88].

Scanning electron microscopy (SEM) is a method of imaging the sample by scanning its surface with a focused beam of electrons. The electrons penetrate into the sample and interact with atoms, producing signals that give information about the surface and morphology of the nanoparticles. As with the SEM method, the electron beam ends on the surface of the sample, so the main disadvantage is the inability to study the internal structure of nanoparticles [75,83,93].

**Figure 6:**
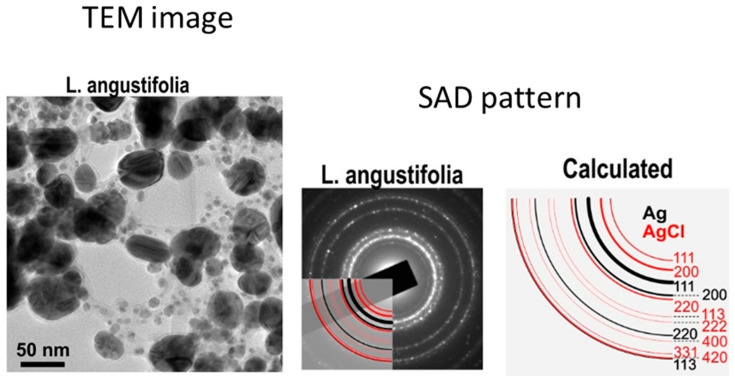
Left: TEM image of AgNPs prepared by *L. angustifolia* from 5 mM AgNO_3_; right: selected area diffraction (SAD) pattern of sample and calculated diffraction rings for Ag (black) and AgCl (red) [61].

Another valuable and frequently used technique is transmission electron microscopy (TEM), which is based on the penetration of an electron beam through a sample, unlike in the SEM method. In this case, we can quantitatively determine a particle’s size, morphology, and size distribution. TEM analysis provides good spatial resolution and also possibilities for various measurements, but the preparation of the sample is very important because this technique requires a thin sample section [75,83]. TEM analysis is often connected with EDX or EDS (energy dispersive X-ray spectroscopy), which provides information about individual elements present in the studied system, as well as with SAD (selected area diffraction), which provides the exact phase composition resulting in the rings or points in the reciprocal space. As an example of TEM analysis, the Ag nanoparticles prepared using L. angustifolia aqueous extract, including the SAD pattern, is in Figure 6 [61].

The diameter of the Ag nanoparticles was 38 nm. SAD patterns as diffraction rings confirmed that the sample contained randomly oriented particles with sizes in the nanometre range [61].

## 4. Interaction of AgNPs with Cancer Therapeutic Modalities

### 4.1. AgNPs and Radiotherapy

Traditionally, radiosensitizers are pharmacologic or chemical agents; however, in recent years, nanoparticles with a high atomic number have opened new prospects for the radiosensitisation of tumours due to their increased probability of producing secondary radiation.

Recent in vivo and in vitro investigations have shown that AgNPs may strengthen cell/tissue radiosensitivity (RT). This method of oncotherapy relies on the interaction of ionising radiation (such as γ -ray, X-ray photons, or charged particles) to kill tumour cells. RT is a conventional adjuvant therapy for a variety of malignancies and is presently offered to around fifty percent of cancer patients [94]. However, its primary disadvantages include tumour-acquired resistance, lack of selectivity, and dosage escalation, which is restricted because of the severe adverse effects of ionising radiation [95,96,97].

The large atomic number of AgNPs is one of the primary characteristics that makes them more appealing as radio sensitizers (Z). Due to the high electron density around the core atom of high-Z elements, ionising radiation might result in an increase in ionisation and the cross-section of biomolecules [98,99]. Similar to other atoms with a high Z-number [80], AgNPs interact with X-ray photons to release secondary electrons. These electrons either interact directly with the DNA, which tends to result in DNA double-strand breaks [100,101,102], or ionise water molecules to produce reactive oxygen species (ROS), which can cause additional damage (such as damage to the DNA, lipid peroxidation, ER stress, and mitochondrial dysfunction) and finally lead to cell death.

The above-mentioned studies provide important evidence that AgNPs are promising agents for improving radiotherapy, not only because the nanoparticles act as radiosensitizers, releasing secondary electrons (increasing the damage of intracellular radiation), but because they also reduce cell proliferation, increase apoptosis, and promote autophagy. In order for AgNPs to be used in clinical settings, more research must be conducted to elucidate the exact processes by which they augment radiation and their impact on various forms of cancer.

### 4.2. AgNPs and Phototherapy

For the treatment of cancer, photodynamic therapy (PDT) and photothermal therapy (PTT) are photo-mediated therapies with distinct modes of action. Both phototherapies are extremely effective and minimally invasive or non-invasive forms of therapy that have gained popularity in recent years. The development of reactive oxygen species, which induce oxidative stress in the case of photodynamic therapy and heat production in the case of photothermal therapy, is responsible for the death of cancer cells induced by the administration of these treatments. These nanoparticle-based medicines reaped major benefits from the development of nanotechnology, which enabled both process tweaking and an improvement in efficacy. The encapsulation of pharmaceuticals, the synthesis of the most diverse organic and inorganic nanoparticles, and the functionalisation of surfaces are some tactics utilised to combine phototherapy with nanotechnology in the pursuit of a successful treatment with minimum adverse effects.

For instance, the surface plasmon response of silver is rather robust when stimulated, which may transform photon energy into hyperthermia for use in PTT [102,103]. Due to its antibacterial properties, silver is often used as an adjuvant agent for wound therapy [104]. Silver tends to be more reactive in environments with a high concentration of O_2_. Ag_2_S, a well-studied nanomaterial containing silver, serves as a suitable carrier for transporting anticancer therapeutic drugs; its use in PTT has also been documented [105,106]. Size-appropriate Ag_2_S is capable of causing a photothermal tumour-killing effect. In addition, it may be used in imaging to offer more precise information for cancer detection [101,102]. In addition, compared to other regularly used photothermal materials, such as gold and copper sulphide, Ag_2_S NPs have superior in vivo stability and safer biological statistics, in addition to high photothermal performance [107]. Therefore, the introduction of Ag_2_S nanoparticles will increase the prospects for cancer PIT treatment.

The work of authors in [108] indicated that BSA/AgNP-loaded hydrogel films may serve as a topical PTT agent that is both effective and safe for the treatment of skin cancer.

### 4.3. AgNPs and Photoimmunotherapy

Combining phototherapy with immunotherapy is recognised as the optimal method for treating both primary and metastatic cancers. Immunotherapy employs a variety of immunological techniques to activate the immune system so it can recognise and eliminate tumour cells. Phototherapy eliminates primary tumours by irradiating them with light, which initiates a sequence of immunological responses by causing immunogenic cancer cell death. Incorporating immunotherapy with phototherapy has led to the development of a unique anticancer technique known as photoimmunotherapy (PIT). This synergistic therapeutic approach may not only increase the efficacy of both medicines, but also transcend their inherent limits, ushering in a new era for cancer treatment.

Recent advances in nanomaterials provide a foundation for PIT. Due to their exceptional physiochemical features, among all these nanomaterials, inorganic nanomaterials stand out as the best PIT mediators. Due to their outstanding drug-loading capacity, inorganic nanomaterials not only serve as carriers to transport immunomodulatory compounds in immunotherapy, but they can also serve as photothermal agents or photosensitizers in phototherapy.

Han et al. [107] created a type of water-soluble Ag_2_S nanoparticle with a particle size of around 15 nm and modified it with the cyclic arginine-glycine-aspartate peptide to increase its tumour penetration and accumulation. Experiments in vivo and in vitro demonstrated that the photothermal effects created by Ag_2_S nanoparticles enhanced the efficacy of cancer therapy, decreased primary tumour recurrence, and relieved metastasis in a model of breast cancer.

As the immunosuppressive TME is an impediment that hampers the efficacy of immunotherapy, Hou et al. [109] designed a hydrogel to encapsulate Ag_2_S QDs inside its hydrophobic core in order to solve this complex problem. DOX and bestatin are hydrophilic anticancer drugs that have been included in this hydrogel. Due to the excellent photothermal performance of Ag_2_S QDs, the sustained release of DOX was triggered to commence in situ vaccination. Bestatin is an immunoadjuvant that may augment the immunological response of the body. After laser irradiation, in vivo findings showed that Ag_2_S QD-based hydrogel could activate the immune response to limit primary tumour development and distal lung metastasis, highlighting the possible future use of Ag_2_S QD-induced PTT in cancer therapy.

### 4.4. Synergistic Interactions Between AgNPs and Chemotherapeutic Medications

**Figure 7:**
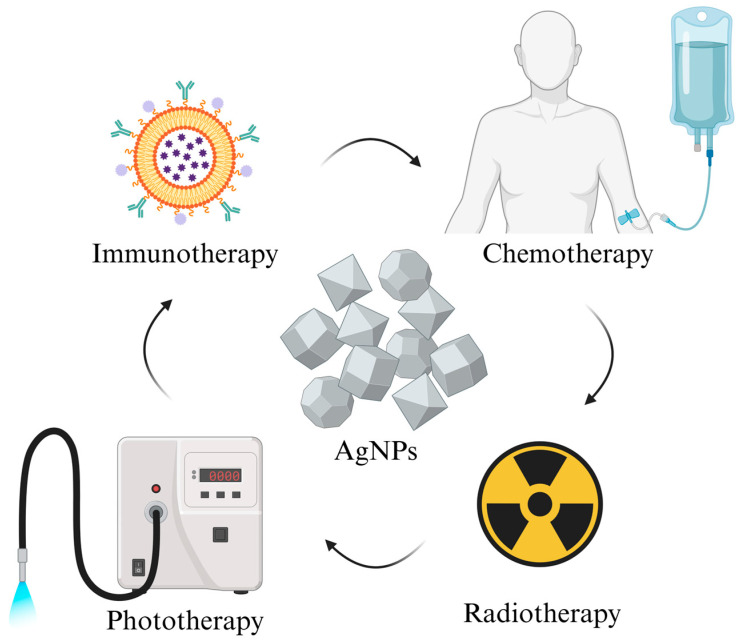
Synergistic interactions between AgNPs and chemotherapeutic medications. Original figure made for review using Biorender software (https://app.biorender.com/ 31 March 2025).

Combination chemotherapy is a treatment that uses two or more medicines that target distinct cancer markers to produce synergistic damage (Figure 7). This method may improve treatment effectiveness and reduce medication resistance and adverse effects [110]. In many circumstances, however, complex dosing regimens, spatiotemporal delivery constraints, and changes in the pharmacokinetic and physicochemical characteristics of the medications may alter the success rates of the therapy [111,112]. Nanotechnology may provide a means for overcoming these limitations. Nanocarrier systems (NCs) are created to carry anticancer medications with clinical approval in order to overcome drug solubility concerns, extend circulation times, and provide controlled drug release. Moreover, NCs may enhance medication accumulation at the tumour location by passive (EPR impact) or active targeting.

Silver NCs may also be used for distribution and function in tandem with cancer small molecule inhibitors. For instance, histone deacetylase inhibitors (HDACis) are a family of medicines that could potentially be used for the treatment of cancer. The action of HDACis may generate several biological effects in cancer cells, including apoptosis and the inhibition of cell growth. In addition, the use of HDACis leads to the hyperacetylation of chromatin, resulting in an open chromatin shape that renders DNA more susceptible to harmful chemicals [113]. Igaz et al. investigated the biological effects of the combination of AgNPs with Trichostatin A, which resulted in a synergistic inhibitory impact on HeLa cell viability, proliferation, and migration [114]. Increased ROS levels and double DNA strand breaks are two potential biochemical pathways linked with these consequences. Gurunathan et al. showed that the combination of AgNPs with HDACis-MS-275 is effective as well. The scientists discovered that the combination therapy results in a significant degree of cytotoxicity owing to cell membrane rupture, oxidative stress, mitochondrial damage, and apoptosis [115]. Shandiz et al. [116] developed a green-synthesized AgNCs systems for imatinib, a kinase inhibitor presently utilised in treatment against several forms of cancer. The researchers showed that the usage of NCs dramatically decreased the viability of MCF-7 cells in comparison to the cytotoxic effects of isolated AgNPs or imatinib. AgNPs are promising agents with the potential to improve combination chemotherapy because they can act as active vehicles for anticancer drug delivery; however, in vivo studies are required to gain a deeper understanding of the systemic outcomes, metabolism, bioaccumulation, and long-term effects of AgNPs combined with anticancer drugs.

Fahrenholtz and colleagues examined the extent of mitochondrial malfunction, apoptotic cell death, and autophagy generated by exposure to AgNPs or AgNPs administered in conjunction with cisplatin using different ovarian cancer cells. They discovered that AgNP treatments were helpful in cell lines with greater intracellular baseline ROS levels but were ineffective in cells with lower starting ROS levels. However, when AgNPs were delivered in conjunction with cisplatin, they lowered the viability of non-AgNP-sensitive ovarian cancer cells [117]. In a separate investigation, synergistic interactions between AgNP and salinomycin were discovered. Salinomycin is an antibacterial drug that is considered to be capable of killing cancer stem cells; hence, it is a prospective option for future chemotherapy [118]. AgNP therapy enhanced mitochondrial failure, autophagy, and apoptosis produced by salinomycin in A2780 human ovarian cancer cells. Individual treatments of AgNP and salinomycin produced ROS production, loss of mitochondrial membrane potential, and caspase-3 dependent apoptosis, which were greatly amplified by combinational exposures [119]. In addition, it has been shown that AgNPs and the topoisomerase I inhibitor camptothecin promote cervical cancer cell death in a synergistic manner. In this work, the combination of AgNPs and camptothecin enhanced ROS levels and produced LDH leakage, mitochondrial dysfunction, and apoptosis, while decreasing antioxidant levels and reducing cell survival and proliferation.

## 5. AgNPs as Drug Delivery Agents

The release of medications at specified target sites in live organisms at a predetermined time is known as drug release timing control. The creation of nanosystems optimised for the release of a medicine at a particular target region often permits the reduction in some of the drug’s undesirable effects and toxicity. Several nanocarriers are now under investigation [120], including organic (liposomes, dendrimers, micelles, etc.) and inorganic (magnetic, silver NPs, gold NPs, quantum dots, etc.) nanoparticles (NPs). In addition to organic and inorganic nanoparticles, there exist hybrid nanoparticles [121] which have an inorganic core surrounded by an organic substance.

A targeted drug delivery system must provide control over the medication’s destiny in the body, therefore preserving non-therapeutic cells and tissues. The nanocarriers of these medications are equipped with tailored and well-defined physical, chemical, and biological features to enhance their cellular absorption or the drug’s relationship with bigger molecular structures [122].

In addition, the ability to control the size, surface charge, and surface chemistry of the nanoparticles acting as carriers, as well as the release of pre-loaded drugs at a specific site, allows conventional therapies to overcome other limitations, such as the need for higher dosages, poor bioavailability, and chemical instability in the administered drug [123]. If the nanocarriers are designed and manufactured to effectively collect on the target, the incidence of systemic adverse effects will be reduced, and the therapeutic effectiveness will increase. Currently, the manufacturing of nanoparticles with predetermined physical–chemical characteristics enables drug-delivery nanoparticles to be adapted to particular diseases and to many kinds of anticancer medications [120,124]. This is because each form of cancer has distinct biological manifestations.

Each nanocarrier carries many drug molecules, which increases the concentration of the medicine effectively delivered to the target tumour without affecting healthy tissues. It is also feasible to transport many anticancer medicines per NP, resulting in an anticancer impact that is synergistic. This permits the concentration of each medication to be decreased, hence preventing toxicity and the development of tumour resistance to chemotherapy. However, the NPs have a limited capacity for drug loading.

The nanoparticle drug delivery systems may be delivered orally, nasally, parenterally, or intraocularly, among other methods, although systemic administration is the most common approach. To enhance patient compliance or minimise production costs, it is feasible to pick one approach over another [125].

Despite their many benefits, nanoparticles (NPs) have constraints that must be addressed before they can be employed frequently in clinical settings or marketed, particularly for targeted delivery of cancer therapies [126]. For instance, fewer than 1% of nanoparticles designed for passive nanodelivery (through the Enhanced Permeability and Retention effect) reach their intended target. Losses are attributed to random dispersion within the tumour and neighbouring tissues, the defensive responses of mononuclear phagocytic cells, and the difficulty of breaching physiological biological barriers. To circumvent some of these obstacles, active cellular nanodelivery techniques provide greater affinity to particular ligands on targets, resulting in a greater likelihood of cellular absorption. Nevertheless, even active targeting relies heavily on passive diffusion throughout the system to reach the target tumour tissues. In addition, the transit of drug-carrying nanosystems via the blood-brain barrier remains a general difficulty [127]. In addition, nanotherapeutic drugs have significant disadvantages, including limited bioavailability, which necessitates the administration of large dosages to compensate for the small number of nanocarriers that effectively reach the target, resulting in severe side effects. Rosenblum et al. [126] provide further information on this issue, including a review of prior clinical studies with nanocarriers for cancer therapies. Consequently, it is necessary to do further research in this area in order to create techniques that mitigate the aforementioned constraints.

Few studies explore the use of AgNPs as drug delivery agents, possibly because of their known toxic effects on cells. However, one study in particular investigated the effect of three NMs (single-walled carbon nanotubes, AuNPs, and AgNPs) on MLO-Y4 osteocytic cells and HeLa cervical cancer cells. When used together with chemotherapeutic agents such as etoposide, there was a significant decline in cell viability compared to the treatment of cells with either NMs or chemotherapeutic agents alone [128]. While the authors proposed the use of NMs in combination with chemotherapeutic drugs for cancer therapy, there was no evidence provided to show a significant differential response between normal and cancer cells to the treatment. In addition, the NMs were administered separately from etoposide or dexamethasone to the cells. The lack of specific conjugation of the drug with the NM makes it difficult to apply this model to in vivo scenarios. Without the specific conjugation of the drug with NM, it is not possible to target the drug to tumour cells only, and normal cells could also be affected by the freely administered chemotherapeutic drug. Table 2 highlights the diversity of silver nanoparticle formulations and their applications in delivering various anticancer drugs, showcasing their versatility and potential in oncology.

## 6. Toxicity of Silver Nanoparticles

### 6.1. Cellular Uptake of AgNPs

Cellular uptake of AgNPs primarily occurs through various forms of endocytosis, including phagocytosis, micropinocytosis, clathrin-mediated endocytosis, and caveolin-mediated endocytosis. The specific pathway utilized is largely determined by nanoparticle size, shape, surface charge, and coating [142]. The internalisation of nanoparticles by cells is governed by their physical and chemical features, including size, shape, surface charge, and composition. In the process of cellular absorption of liposomes, quantum dots, gold, and silica nanoparticles, it has been shown in recent years that particle size is a crucial element. Previous studies suggest that smaller NPs are imported into cells by endocytosis or diffusion, whereas bigger NPs are imported via phagocytosis [142,143,144]. Larger-sized nano silver may be too big to be absorbed and may stay on the surface, where it may activate several receptor-mediated signalling processes or induce lipid peroxidation. Nanoparticles less than 100 nm in diameter have greater cytotoxic activity and greater ability to escape from a mononuclear phagocytic system [145,146]. Gliga et al. [147] recently demonstrated that the cellular absorption of silver was markedly greater when A549 and BEAS-2B cells were exposed to silver nanoparticles compared to silver ions. Consequently, there is emerging evidence supporting the ‘Trojan horse’ hypothesis, which posits that the particle facilitates the uptake of AgNPs through endocytosis, thereby enhancing the intracellular bioavailability of silver. Several prior investigations have concentrated on examining the size-dependent effects of AgNPs. Conversely, Liu et al. [148] indicated that 5 nm AgNPs exhibited greater toxicity than 20 nm and 50 nm particles across four distinct cell lines (A549, HepG2, MCF-7, and CGC-7901), while Kim et al. [149] demonstrated an increased release of LDH and diminished cell viability with 100 nm AgNPs compared to smaller particles (10 nm and 50 nm). Overall, there is limited research that has examined size-dependent effects and underlying mechanisms employing well-characterized AgNPs.

### 6.2. Biodistribution of AgNPs

The potential and degree of in vivo toxicity of AgNPs are principally determined by their pharmacokinetic properties and target organ dosimetry [133,134]. It has been established that the pharmacokinetics of AgNPs vary depending on dosage, exposure route, species, and gender [150]. Citrate-coated 7.9 nm AgNPs, for instance, exhibited a plasma half-life of 99 h (1 mg/kg) after IV injection and 30 h (10 mg/kg) after oral exposure in rats [151], but in rabbits, following IV treatment, the half-lives were 11.7 and 16.3 days in the 5 and 0.5 mg/kg groups, respectively [152]. The fact that the half-life of citrate-coated 7.9 nm AgNPs is three to four times shorter in rats compared to rabbits may be partially explained by the longer transit time in the systemic circulation of larger animals compared to smaller rodents, which modulates the extent of nanoparticle biocorona formation, as predicted by pharmacokinetic models [153,154]. Intriguingly, the half-life after an IV injection of 120 mg/kg 90.5 nm AgNP agglomerates was 15.6 h in male mice and 29.9 h in female mice, demonstrating a gender difference.

The bioavailability of AgNPs is typically poor and is dependent on particle size, dosage, surface coating, and soluble fraction. Following a single oral administration to rats, the bioavailability of citrate-coated 7.9 nm AgNPs was 1.2% for 1 mg/kg and 4.2% for 10 mg/kg [151]. For cutaneous exposure, research has found that 25 nm AgNPs penetrated both intact and injured in vitro human skin with a low flux compared to the absorption rate of metal particles. TEM revealed the presence of AgNPs in the stratum corneum and outermost surface of the epidermis, but not in the dermis, indicating that a minor portion of the AgNPs dissolved and diffused through the skin layers as elemental Ag. The metabolism of AgNPs after oral exposure is as follows: AgNPs may be ionised in the stomach to create Ag^+^; however, the dissolution is partial because of the short gastric residence duration (10–240 min). Ag^+^ and other soluble complexes [AgCl, AgCl^2−^, and AgCl_3_^2−^] may be absorbed through the gastrointestinal tract into systemic circulation, where Ag^+^ can bind to thiol-containing proteins such as serum albumin and tiny thiol molecules such as glutathione (GSH). These complexes create H^+^ and GS-Ag, which are subsequently converted into Ag-GSH complexes and distributed throughout the body. Ag-thiol complexes are converted to AgNPs by UV photodecomposition, or by visible light at a slower pace. In addition to thiols, AgNPs may be sulfidated to produce Ag_2_S-NPs, which can then react with selenium (Se) to produce Ag_2_Se-NPs and Ag/S/Se argyrial secondary particulates, but not the main particles.

**Figure 8:**
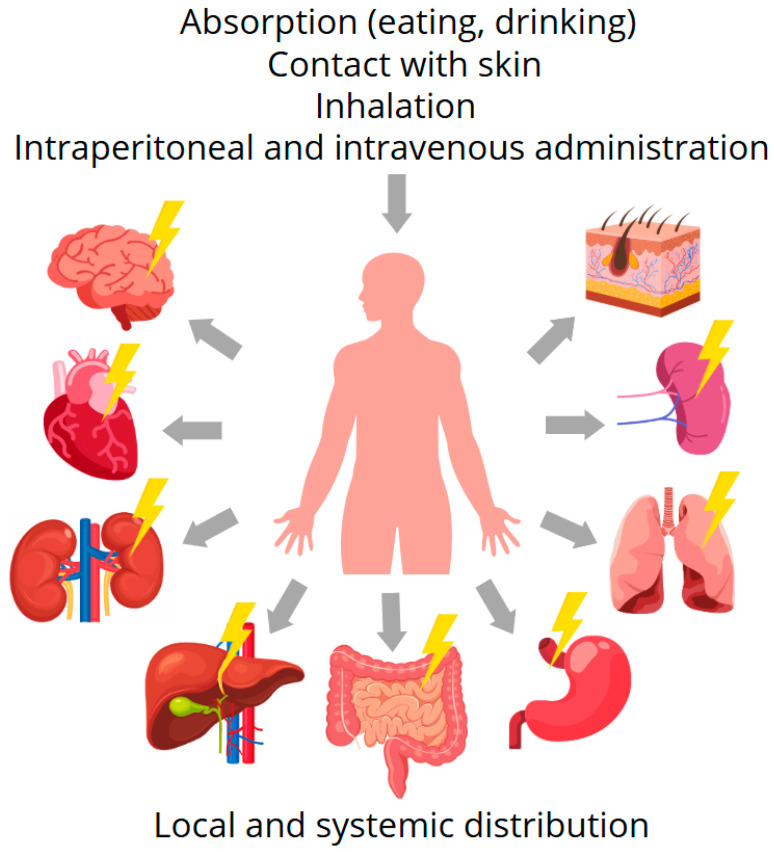
Biodistribution of AgNPs in living organisms [139,140,141,142]. Original figure made for review using Canva software (Version 1.106.0, with the latest update on 8 April 2025).

The biodistribution of AgNPs is size-dependent and predominantly targets the liver, followed by the kidneys, spleen, and other organs. While these organs are commonly involved, the specific distribution profile also depends on the route of exposure [150]. Several cell types, including Kupffer cells, hepatocytes, and sinusoidal endothelium cells, have accumulated AgNPs in the liver. AgNPs were found in all areas of the brain, medulla, inner medulla, and cortical glomeruli in the kidney [139] (Figure 8). The distribution of AgNPs greater than 80 nm in the spleen has been demonstrated to be high [155]. Following oral administration, AgNPs of up to 110 nm may be transported to and stay in the brain for two months [156].

**Figure 9:**
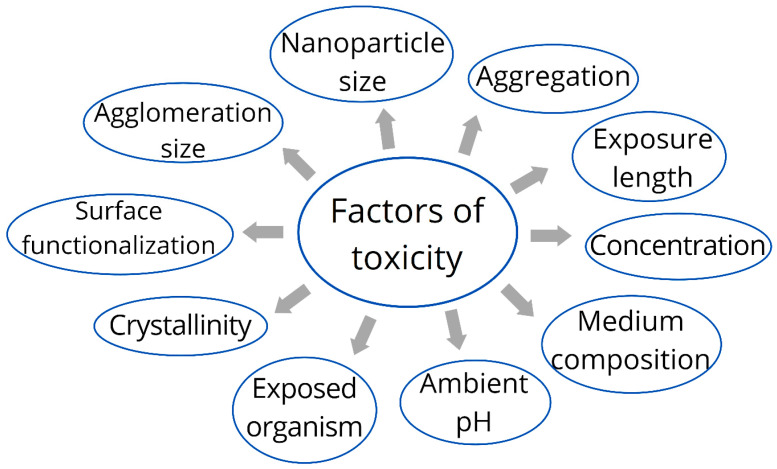
The main factors of toxicity of AgNPs. Original figure made for review using Canva software.

Individuals are increasingly likely to be exposed to AgNPs as the use of nano silver-containing goods increases. Generally, the biodistribution and accumulation of AgNPs vary significantly depending on the route of exposure. For instance, following dermal exposure, AgNPs tend to localize in the epidermis and rarely penetrate to the dermis unless the skin barrier is compromised. Inhalation leads to deposition primarily in the lungs, followed by translocation to the liver and brain in some cases. Oral exposure results in low bioavailability, with predominant accumulation in the liver and gastrointestinal tract. Intraperitoneal and intravenous routes, however, result in a more systemic distribution, with the liver, spleen, and kidneys being the primary organs of accumulation due to mononuclear phagocyte system uptake. These patterns highlight the importance of exposure route in determining AgNP pharmacokinetics and potential toxicity. The liver, lung, spleen, and kidney are the primary organs for nano silver accumulation in living organisms [142,143,157,158]. As illustrated in Figure 9, the identified determinants of toxicity include nanoparticle size, aggregation, exposure length, agglomeration size, medium composition, ambient pH, crystallinity, surface functionalisation, concentration, and the organism exposed [159]. Due to the large number of variables involved, it is impossible to anticipate the toxicity of each nanoparticle; consequently, thorough testing is necessary for each NP.

As a consequence, the particles may be taken up by cells, posing the risk of contact with biological macromolecules inside the cell. The correlation between the uptake mechanisms and cellular distribution of AgNPs and different cytotoxic effects is direct. Therefore, it is essential to investigate the mechanisms of cellular absorption and the intracellular activities of exogenous AgNPs, which will enable us to better comprehend their biological impacts and maximise their medicinal uses.

Although nanotechnology has grown more relevant in recent years, there is currently little and unconfirmed evidence about the short- and long-term toxicity hazards of exposing people, animals, and the environment to NPs, especially silver NPs [160,161]. The production of NPs might necessitate the usage of compounds that are harmful to live creatures or the environment. Most studies only examine the effects of NPs when they are inhaled, causing damage to the respiratory system. In animal models, metal NPs have shown an increase in the production of radical species and impairment of platelet function [162], among other harmful consequences.

In vitro investigations have established that AgNPs are hazardous to several organs, including the lungs, liver, brain, vascular system, and reproductive system. In this context, AgNPs would stimulate the production of genes involved in the development of the cell cycle and apoptosis.

Possible harmful pathways include the induction of reactive oxygen species (ROS) and oxidative stress, leading to DNA damage and apoptosis. De Matteis et al. [163] concluded that the toxicity in cells treated with AgNPs is mostly produced by the release of Ag+ ions in the cytosol, after endocytosis and breakdown of the AgNPs in an acidic environment. Therefore, the oxidative stress, DNA damage, and cell death seen in the presence of AgNPs are mostly attributable to the disruption of natural metabolic and cell cycle mechanisms caused by silver ions in the cytosol. This idea is strengthened by the activation of metallothioneins and the avoidance of cytotoxicity by Ag+ chelating agents. In contrast, Suarez et al. [164] aimed to comprehend the physiological disturbances induced in hepatic cells by exposure to extremely low concentrations of silver nanoparticles, simulating chronic exposure. The rapid entrance of soluble silver ions into the nucleus, where it accumulates and affects nuclear receptor activation, is deleterious to liver metabolism, according to the scientists.

In vivo investigations on the cytotoxicity and genotoxicity of AgNPs have also been conducted. Due to their small size and ability to be transported by air, water, or physical contact, AgNPs can enter the human body through multiple exposure routes, including inhalation, dermal contact, ingestion, and injection. AgNPs may readily move from the site of exposure to other organs, entering cells along the way [160]. Lee et al. discovered that exposure to AgNPs modifies the expression of many genes linked with motor neuron problems, neurodegenerative illnesses, and immunological function, suggesting the potential neurotoxicity and immunotoxicity of exposure. In a separate study, Wang et al. [165] determined that AgNPs induce reproductive failures, abnormalities throughout development, and morphological deformities in some animal models.

Several physicochemical properties, such as concentration, charge, surface functionalisation, size, and shape, impact the genotoxicity and cytotoxicity of AgNPs [166,167,168]. The experimental data published until recently are insufficient to precisely determine the harmful effects and processes of AgNPs. However, toxicity restricts its in vivo use [169,170].

For AgNPs, the utilisation of cell-derived membranes and extracellular vesicles (EVs) as intelligent and biocompatible nanoparticle coverings is mostly unexplored. However, as shown by Zhao et al. [171], this method has immense potential to overcome the clinical limitations of AgNPs. In their work, the scientists coated AgNPs with FA-functionalized red blood cell membranes, and the system demonstrated excellent biocompatibility, tumour targetability, and anti-lymphoma characteristics in both in vitro and in vivo settings. The scientists discovered no in vivo adverse effects caused by the system [172]. Given the immense potential of CM-NP in oncotherapy, more research is urgently required, as it might increase AgNP’s tumour selectivity and capacity to overcome biological barriers.

Nanoparticles, particularly AgNPs, possess a remarkable surface-to-volume ratio, resulting in a high level of reactivity with other chemicals and biomolecules. AgNPs are capable of entering cells and forming a protein corona inside. This may interfere with the function or activity of intracellular proteins [172,173]. In reality, it is not anticipated that the interaction between AgNPs and biomolecules would result in significant cytotoxicity, such as cell death. However, the impact generated by AgNPs might vary depending on the kind of cells exposed to them. For instance, in reproductive cells, the interaction between AgNPs and hormones seems to interfere with cell or organ function. Since it has been demonstrated that nanoparticles can interact with biomolecules, such as natural or synthetic hormones like 17-estradiol and 17-ethinylestradiol [174,175], it would not be an exaggeration to say that internalised AgNPs in cells may interact with natural hormones and disrupt normal hormone–receptor interaction.

### 6.3. Principal Pathways of AgNPs’ Toxicity

The toxicological mechanism of AgNPs primarily stems from their capacity to generate substantial quantities of reactive oxygen species (ROS), which can compromise the integrity of the cell membrane and induce apoptosis [159,176,177]. AgNPs readily oxidise in the presence of O_2_ and other molecules [178], resulting in the formation of Ag+ [179]. Increased production of reactive oxygen species (ROS) and high ROS levels induce oxidative stress [180,181]. Endoplasmic reticulum stress functions as a cell signal transduction system and a crucial defence mechanism [182], inherently connected to oxidative stress [183]. Endoplasmic reticulum stress is a protective response aimed at diminishing the concentration of unfolded proteins within the cell to avert their aggregation [184]. AgNPs can disturb endoplasmic reticulum (ER) homeostasis [185], impair proper protein folding, and result in the accumulation of unfolded and misfolded proteins within the endoplasmic reticulum lumen, hence producing excessive ER stress [186]. Certain experiments have demonstrated that AgNPs can impair mitochondrial functioning via non-ROS mechanisms [187]. In response to research on the probable toxicity mechanisms of AgNPs, we have examined five primary mechanisms of AgNPs toxicity (see Table 3).

### 6.4. Organ Specific and Cellular Toxicity

The biological system can absorb nano silver in a number of ways. The routes of exposure, along with factors such as time, size, state of aggregation, and dose of silver nanoparticles, are interconnected with their bioavailability, biodistribution, and associated pathological symptoms.

Various animal models are established and utilised to investigate the toxicity of nano silver to organs (see Table 4).

As was explained earlier, the potential mechanisms of AgNPs’ toxicity at the cellular level include mainly oxidative and non-oxidative stress pathway induction, ultimately leading, in many cases, to DNA damage, mitochondrial impairment, and cell death. Even very low doses of nano silver may trigger structural or functional damage to target cells [201]. In Table 5, the toxic effects of nano silver, including mechanisms in selected human cell lines, are presented.

## 7. Standardized and Alternative Safety Assessment Methods for AgNPs

To accurately assess the safety and effects of a nanomaterial, it is essential to comprehend its fundamental physicochemical qualities and its interactions with living cellular systems. This section presents the predominant methodologies for characterising nanomaterial properties and a series of in vitro toxicity assessments employed to evaluate material safety. The methodologies outlined below do not encompass the complete range of available instruments and techniques but rather provide a sufficiently comprehensive preliminary evaluation for assessing a new nanomaterial. Assessment techniques include in vitro assays, standard regulatory toxicology tests, and alternative models.

### 7.1. In Vitro Assays

To evaluate the safety and behavior of nanoparticles in biological systems, a variety of classical analytical methods are employed. The table below summarizes key techniques, their purposes, underlying principles, and representative references commonly used in nanoparticle research (Table 6).

The genotoxicity of silver nanoparticles was assessed using the alkaline comet assay in human peripheral blood cells [217]. Following a 3 h exposure, the results indicated that AgNPs (50 and 100 g/mL) caused DNA damage. Additionally, a brief exposure of 5 min also indicated DNA damage. In conclusion, the study has shown that the synthesised AgNPs caused DNA damage in human peripheral blood cells, as evidenced by the alkaline comet assay. Furthermore, the results indicated that there was no evidence of DNA damage induced by hydrogen peroxide when the cells were exposed to Ag nanoparticles.

Li et al. [219] employed 5 nm AgNPs to assess their genotoxicity through an in vitro micronucleus assay. The exposure to Ag nanoparticles resulted in an increased frequency of micronuclei, with the extent of this increase being dose-dependent on the AgNPs administered. At a concentration of 30 μg/mL (with a relative population doubling of 45.4%), AgNPs resulted in a notable 3.17-fold increase, reflecting a net increase of 1.60% in micronucleus frequency compared to the vehicle control, indicating a weak positive response according to the study’s criteria. The results indicate that 5 nm AgNP exhibit genotoxic effects on TK6 cells.

### 7.2. Standard Regulatory Toxicology Tests

To ensure the safety of nanomaterials, regulatory agencies such as the FDA, EMA, and OECD recommend standardized toxicological testing. In vivo AgNP testing studies are summarized in Table 7.

### 7.3. Alternative Models

Avian embryos play an important role in basic medical research. This alternative animal model is cost-effective, readily available, reproducible, and reliable. This model meets the requirements of the 3Rs—Reduce, Refine, and Replace as outlined by Russell and Burch (1959) [224]. They aim to reduce animal suffering, pain, and distress, to replace living organisms, and to minimize the number of animals used for testing. Chicken embryos are the most commonly used, but studies can also be carried out on quail [225] or ostrich embryos [226,227].

The bird embryo model does not need approval from the Ethics Committee for Animal Experimentation because it is exempt from the legislation on the protection of animals used for scientific purposes (2010/63/EU) as well as from the laws in force in the United States. Research suggests that chicken embryos do not experience pain until approximately day 13 of incubation [228]. Therefore, in most countries, the chicken embryo is not used after this day. Slovakia is an exception, where the chicken embryo can be used up to the day of hatching.

The chorioallantoic membrane (CAM) is a simple and richly vascularized extraembryonic membrane that surrounds the developing avian embryo [229]. It serves as a storage compartment for nitrogenous waste. It has the function of transporting calcium for bone formation and facilitating respiration by transporting carbon dioxide and oxygen [230]. From a scientific perspective, the CAM model has been used to analyse irritant potential [231], angiogenesis [232], the biocompatibility of materials [225] and tumour implantation [233]. The CAM in chickens is an inherently immunodeficient host, which allows the formation of xenografts [226]. In nanotoxicology research, the in ovo model is useful in assessing the toxicological impact of nanoparticles by offering better CAM surface accessibility and better visibility in studying the effects of nanomaterials [234]. In addition, the CAM model serves as an excellent tool for assessing the biocompatibility and angiogenic responses of biomaterials, providing insights into their potential applications in tissue engineering and regenerative medicine [235].

**Figure 10:**
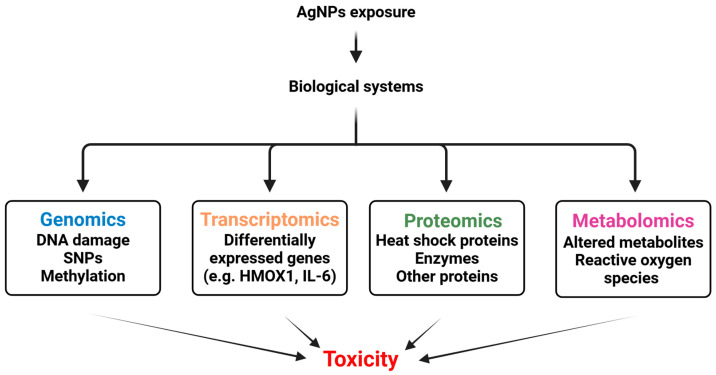
Multi-omics and AgNP safety profile assessment. Original figure made for review using Biorender software.

Silver nanoparticles exhibit antibacterial activity; therefore, they can be applied topically for the treatment of bacterial diseases on the skin or on the ocular or mucosal membrane. In the case of optical, nasal, vaginal, or oral application, an irritancy test must be performed [236,237]. The in ovo test helps to develop a safety profile for the use of biosynthesized AgNPs [238]. The irritation potential and safety of AgNPs applied in solution or incorporated into semi-solid/solid dosage forms with regards to mucosal surfaces can be monitored using the Hühner-Embryonen Assay on the chorioallantoic membrane of the hen embryo (HET-CAM) (Figure 10). It is among the recommended test methods of the National Institutes of Health (NIH) and its division, the Interagency Coordinating Committee on the Validation of Alternative Methods (ICCVAM) (Recommended Test Methods, NIH Publication No. 10-7553—2010). It was developed as a rapid, sensitive, and inexpensive test of the irritant potential of substances, using hens. The test evaluates the negative effect (vessel lysis, hemorrhage, and intravascular and/or extravascular coagulation) of the administered substance on the chorioallantoic membrane. The irritability score is determined using a numerical value of the individual effects as a function of time [237]. Local toxicity of substances can be attributed to several factors including chemical irritation, non-physiological pH, osmolarity, inappropriate salt concentration, or toxicity to cells [239]. The irritant effect after topical application leads to unwanted pain and inflammatory processes, with subsequent tissue damage [240].

The irritation test has been used for formulations that are intended to be applied to vaginal [241], nasal [239], or oral mucosa. The HET-CAM test has also been applied to other formulations intended for the treatment of oral mucosal diseases. Specifically, various oral products have been tested, including dental adhesives [242], sublingual nanocapsules [243], and hydrogels designed for the treatment of oral carcinoma [244,245]. The HET-CAM model replaces the Draize rabbit eye irritation test [246] so that the results can be applied to formulations intended for ocular administration in ophthalmology [247].

Sarau et al. (2024) observed the irritant in ovo potential of AgNPs prepared using the ethanolic extract from the peel of *Punica granatum* L. They found that a lower concentration of AgNPs (10 µg/mL) caused haemorrhage and vessel lysis when applied to CAM and was classified as a moderately irritant compound. On the other hand, the higher concentration of 50 µg/mL was classified as a weak irritant as only mild coagulation was observed. The results of in ovo toxicological screening show that a higher concentration of AgNPs has a better safety profile compared to a lower concentration [238].

Contradictorily, no irritant effect of silver nanoparticles biosynthesised using *Berberis vulgaris* L. fruits was observed [248]. Berberis vulgaris L. fruit extract, with 5.5 mM AgNO_3_ and AgNPs, did not show any of the three processes (vessel lysis, haemorrhage, and coagulation) that determine irritant potential. In the case of AgNPs, a little vasoconstriction was visible after 2 min. After 5 min, however, the vessels returned to their pre-application state. However, vasoconstriction is not considered an irritant effect according to Luepke (1985) [237]. Overall, the results show that application of Berberis vulgaris L. extract, with 5.5 mM AgNO_3_ and AgNPs prepared from it, does not cause any irritation to vessels or their surroundings [248].

## 8. Novel Methods and Approaches Evaluating AgNPs’ Toxicity

As AgNPs attract increasing interest due to their widespread use in medicine, consumer products, and industrial processes, their potential toxicity requires the development of new evaluation methods and procedures beyond traditional toxicity testing. This need is driven by the unique physicochemical nature of AgNPs, their complex interactions with biosystems, and their relatively long persistence in the environment. AgNPs have unique properties, such as high surface area to volume ratio, reactivity modulated by the size of the NPs, and variable dissolution rate, which tend to have a significant impact on their toxicological activity [165]. Unlike bulk silver, AgNPs have the ability to generate reactive oxygen species (ROS), induce DNA damage, and cause oxidative stress at the cellular level [249]. The release of Ag+ ions by NPs also complicates toxicity analysis, as both nanoparticles and single ions are responsible for bioactivity [250].

There are several limitations in conventional toxicity assessment methods, such as in vitro cytotoxicity assays (e.g., MTT, LDH) and in vivo animal assays, for AgNPs. Most standard assays can interfere with nanoparticles and the signals they generate, and therefore may provide significantly inaccurate results [251]. In vivo experiments, although informative, are expensive, time-consuming and raise ethical concerns. Furthermore, environmental toxicity studies may not truly assess the long-term effects of AgNPs due to their aggregation, transformation, and accumulation in the environment [252].

The toxic mechanisms of AgNPs are complex and depend on particle size, shape, coating, and duration of exposure. AgNPs have been shown to cause inflammation, mitochondrial disruption, and genotoxicity depending on the cell type or organism [253]. In addition, they penetrate biological membranes and physiological barriers, which complicates risk assessment.

New approaches with advanced analytical tools and high-throughput screening are needed to overcome the limitations of traditional methods. Omics technologies such as genomics, proteomics, and metabolomics provide comprehensive data on toxic interactions at the molecular level [254]. Machine learning and predictive models based on artificial intelligence improve risk assessment by analysing large data sets and identifying toxicity patterns [255]. In addition, microfluidic devices and organ-on-a-chip models allow for real-time dynamic toxicity assessment that more closely resembles physiological conditions [256].

Since traditional assays are unable to monitor and evaluate real-world exposure conditions, such as chronic low-dose exposure and bioaccumulation effects, new in vitro 3D models, co-culture systems, and environmental monitoring techniques provide more relevant toxicity data [257]. In addition, green toxicology approaches, which focus on developing safer nanomaterials with minimal environmental and health impacts, are increasingly being integrated into regulatory frameworks [258].

In the following text, we provide a comprehensive overview of the latest findings from the aforementioned methods and approaches.

### 8.1. Multi-Omics and AgNPs Safety Profile Assessment

#### 8.1.1. Genomics

Genomics has been utilised to improve science and research and customise therapeutic care since the Human Genome Project was finished in 2001. Finding out how genes are structured, function, and change is the main goal of genomics. It is a new field of study and medicine that uses a person’s genetic information to improve health outcomes and clinical care [259]. As set out by the National Human Genome Research Institute (NHGRI), genomics encompasses the investigation of direct data regarding DNA and RNA, with the exception of downstream derived products (e.g., proteomics, metabolomics). DNA sequences record genomic variation at the chromosome level, including structural variations (SVs), and at the single nucleotide level, including single nucleotide polymorphisms (SNPs). Genomic variation resulting from alternative splicing events or gene expression is captured by RNA sequences [260].

Given the significant toxicity of AgNPs, the use of genomics represents a useful tool [252]. Studies have produced results describing the impact of AgNPs on the genome at different levels of organisms or organ systems. A study by Gokulan et al. found that while short-term exposure (24 h) to 10 nm AgNP had minimal effect on selected viruses, long-term exposure (7 days) led to a significant reduction in viral/phage populations. Whole-genome sequencing and bioinformatics analyses revealed that AgNP exposure affected several intestinal bacteriophages associated with bacterial genera such as *Enterobacteria, Yersinia*, and *Staphylococcus*. The findings suggest that small-sized AgNP could disrupt the gut microbial ecosystem by inactivating resident phages essential for gastrointestinal health [261]. AgNPs are known for their antimicrobial activity, but their effects on the eukaryotic genome are less well known. Using the model organism *Schizosaccharomyces pombe* (fission yeast), which shares ~23% of its genes with humans, and a combination of mutation accumulation (MA) experiments, whole-genome sequencing, and RNAseq transcriptomics, they found that the base-pair substitution (BPSs) mutation increased 3.46 times. These mutations were particularly enriched in coding regions (i.e., gene sequences) and also significantly increased G:C→T:A transversions, a known signature of oxidative DNA damage. In addition, AgNP-treated yeast showed downregulation of genes related to DNA repair (e.g., pms2, rad22), cell membrane and nutrient transport, and energy metabolism [262]. Mutagenicity was also evaluated in mice and humans. A subtoxic concentration of 1.0 µg/mL of 20 nm citrate-coated AgNPs was used for 24 h on L5178Y TK+/− mouse lymphoma cells. AgNPs induced low-level mutations, increasing their frequency 1.12-fold compared to control, and the mutation frequency increased 4× with short-term exposure, likely due to mutations occurring in fewer cell divisions. The mutation types caused by AgNPs were predominantly at G:C base pairs, mainly G:C→T:A transversions and G:C→A:T transitions. The authors noted more tandem mutations (two adjacent base changes), mainly involving G or C bases. The study also used 4-NQO (4-nitroquinolone-1-oxide), a known mutagen, as a positive control. 4-NQO caused much stronger mutagenic effects (a 130-fold increase in mutation rate) [263].

RNA-seq was performed on murine MC3T3-E1 pre-osteoblasts after exposure to 20 µg/mL for 24 h, followed by 24 days of differentiation. AgNPs were able to significantly alter the expression of some genes. The upregulated genes were mainly Bmp6, Fosl1, and Sstr5 (showed a 61× increase), but some toxicity- and metabolism-related genes (e.g., Gsta1–4, Cyp1a1, Mt1/2) were also strongly upregulated, suggesting an oxidative stress response [264].

AgNPs similarly induced DNA damage in Chinchilla lanigera bone marrow cells at three concentrations (5, 10, and 20 µg/L) for three durations (3, 6, and 24 h). DNA damage was detected using the comet assay (single-cell gel electrophoresis). A higher level of genomic instability was observed at a concentration of 10 µg/L [265].

Lu et al. studied developmental toxicity in zebrafish embryos (4 hpf) exposed to AgNPs with a size of 10–100 nm, with an average size of ~51.7 nm, at concentrations of 0, 1, 2, and 4 mg/L for 72 h. The results showed reduced survival rate, hatching, body length, and weight and an increased number of malformations (e.g., spinal curvature, pericardial edema). Thousands of genes were significantly altered in a dose-dependent manner. This mainly involved genes for DNA replication signalling pathways (e.g., pcna, pola2, mcm2), cellular senescence (e.g., tgfb2, e2f1, cdk4), oxidative phosphorylation, and ATP and protein binding. Among other things, mitochondrial dysfunction occurred (complex I–V activity decreased with increasing AgNP dose and most mitochondrial respiratory chain-related genes were downregulated).

This effect was associated with oxidative stress (increased ROS and malondialdehyde production), alterations in the expression of enzymes such as SOD and CAT, and the activation of apoptosis [266].

Genomics tools like microarray analysis and RT-PCR were used to study the effect of AgNPs after inhalation, their effect on gene expression in the mouse brain (specifically in the cerebrum and cerebellum), and to identify potential biomarkers of exposure and toxicity. Male C57BL/6 mice were exposed to AgNPs (22.18 nm size) for 6 h/day, 5 days/week over 2 weeks using a nose-only inhalation system. A total of 468 genes in the cerebrum and 952 genes in the cerebellum related to motor neuron development (Rock1, Als2, Prkcd, Kif5c, and Prkg1), neurodegenerative diseases (Aqp1 and Tac1), and immune system functions (Hivep2, Malt1, and Sema7a) showed significant changes in expression (*p* < 0.05). Some brain-expressed genes also showed consistent changes in whole blood, pointing to their potential as non-invasive exposure biomarkers (e.g., Arg2, Ifit1, Rabep1) [267].

In addition to genomic changes, AgNPs can also induce epigenetic changes. In a study on HEK293T (human embryonic kidney cells) using 5-mC quantification (to assess overall DNA methylation levels), MeDIP-seq (to identify methylated DNA regions), and RNA-seq (to measure gene expression), the authors found that 25 nm PVP-coated AgNPs caused DNA methylation changes, primarily affecting gene coding regions, and also altered methylation enzyme levels, especially DNMT1 and DNMT3A. A total of 12 genes were upregulated and hypomethylated and 22 genes were downregulated and hypermethylated, which may indicate a correlation between methylation and expression [268].

#### 8.1.2. Transcriptomics

Transcriptomics is the study of the structure, function, and evolution of the transcriptome (i.e., the entirety of RNA transcripts produced by the genome) of a given organism or community of organisms under a variety of conditions [269]. Transcriptomic studies show that silver nanoparticles (AgNPs) cause significant changes in gene expression related to oxidative stress, death, DNA damage, and inflammation. Essential systems comprise the activation of stress response genes (HMOX1, SOD2), pro-apoptotic markers (BAX, CASP3), and inflammatory cytokines (IL-6, TNF-α). The effects often depend on dosage, time, and size, which emphasises the need of transcriptomics in evaluating AgNP toxicity [270]. Piersanti et al. (2021) employed RNA-seq to investigate the transcriptomic responses of Tetrahymena thermophila to casein-coated AgNPs (~30 nm) at sublethal doses and compared them to AgNO_3_ exposure. AgNPs significantly increased the number of differentially expressed genes (DEGs), which included oxidative stress markers such as GPX, thioredoxin, and glutathione S-transferases. Phagocytosis, nitrogen metabolism, and protein phosphorylation were among the distinctive AgNP-specific responses. The dysregulation of critical genes, including CDK6, BOP1, and RPT6, suggests that the cell cycle and proteostasis were affected. These transcriptomic insights elucidate particle-specific toxicity that extends beyond the effects of dissolved Ag⁺ [269]. In another study, the effects of approximately 55 nm myricetin-synthesized silver nanoparticles (AgNPs) on the gene expression of NIH3T3 mouse embryonic fibroblast cells were evaluated by RNA-seq. A total of 176 upregulated and 136 downregulated genes were identified by transcriptomics, including HMOX1, HYAL1, HIST1H2BN, and HIST1H3A, which were pinpointed by broad dysregulation. AgNP-induced oxidative stress, mitochondrial dysfunction, DNA damage, and apoptosis were accompanied by p53, p21, and caspase-3 activation. The importance of this also extends to the fact that AgNPs had an effect on epigenetic processes such as nucleosome assembly and histone gene expression [115]. Using single-cell RNA sequencing (scRNA-seq) and mass cytometry, the transcriptomic responses of human immune cells to 40 nm polyethyleneimine-coated silver nanoparticles (bPEIAg40) were investigated. With the internalisation of the majority of AgNPs by monocytes and B cells, AgNP uptake was cell-type dependent and controlled by gene expression. Transcriptomic analysis revealed B cells upregulating NRF2-related oxidative stress genes, while monocytes demonstrated the activation of Fcγ-mediated phagocytosis and repression of HLA-related immune genes [271]. When human lung epithelial BEAS-2B cells were exposed to 10 nm silver nanoparticles (AgNPs) at a concentration of 1 µg/mL for six weeks, transcriptome changes were investigated. It was revealed that 1717 genes were differently expressed, of which COL1A1, MMP2, and TGFβ 1 were increased. This shows that pro-fibrotic and epithelial-mesenchymal transition (EMT) pathways were activated. Transcriptomics revealed widespread gene expression reprogramming associated with fibrosis and potential transformation, despite minimal change in DNA methylation. Such findings demonstrate the applicability of transcriptomics for predicting the ultimate molecular effects of low-dose AgNP treatment [272].

#### 8.1.3. Proteomics

Proteomics is the large-scale study of proteins, the functional molecules in cells that regulate virtually all biological processes. Unlike the genome, the proteome is dynamic, changing with time, environment, and disease state [273]. By identifying and quantifying proteins, proteomics provides critical insights into cellular function, disease mechanisms, and biomarker discovery [274]. Advanced techniques such as mass spectrometry have made proteomics a cornerstone of systems biology and precision medicine [275].

Using an integrative proteomic and metallomic strategy, the mechanism of hepatotoxicity was studied, and it was found that polyvinylpyrrolidone-coated silver nanoparticles (diameter ~20 nm) have a significant effect on protein composition. The study identifies six key proteins (glutathione S-transferase, peroxiredoxin, myosin, elongation factor 1, 60S ribosomal protein, and 40S ribosomal protein) that interact with AgNPs, including glutathione S-transferase (GST), myosin, and ribosomal proteins, whose interaction is associated with oxidative stress and cell death [276]. However, citrate-coated AgNPs (Cit-AgNPs, 20 nm and 60 nm) induce distinct hepatotoxic mechanisms in human liver cancer (HepG2) and normal (L02) cells. Transcriptomic validation confirmed the upregulation of MT1F, MT1G, HMOX1, and PPP1CC and the downregulation of PDHA2, highlighting oxidative stress, mitochondrial dysfunction, and detoxification pathways. Smaller AgNPs induced more pronounced effects and transcriptomics confirmed dysregulations at the protein level [277]. In addition, silver nanoparticles derived from cotton leaf extract (AgNPs, 13–40 nm) were able to induce the intrinsic pathway of apoptosis in A549 lung cancer cells. Using semiquantitative RT-PCR, upregulation of Bax and p53 and downregulation of Bcl-2 were revealed, confirming transcriptomic modulation of apoptotic genes. AgNPs disrupted mitochondrial membrane potential, leading to cytochrome c release and caspase-3/9 activation [278]. Several studies have compared the mechanisms of gill toxicity in common carp exposed to 12 ± 3 nm PVP-coated AgNPs to silver nitrate. Although transcriptomics was not directly applied, proteomic findings correlated well with previous transcriptomic studies, showing altered expression of genes associated with the cytoskeleton, oxidative phosphorylation, and VEGF signalling. In particular, AgNPs upregulated cytosolic phospholipase A2 and collagen type I alpha while downregulating ATP synthase subunit α, indicating the disruption of energy and vascular pathways at the transcriptomic level [279]. A similar study investigated the mitochondrial toxicity of PVP-coated AgNPs (~16 nm) in carp gills using proteomics, revealing 362 differentially expressed mitochondrial proteins. Although transcriptomics was not directly applied, previous studies by the same authors reported AgNP-induced downregulation of ACO and IDH3, key TCA cycle genes, supporting transcriptomic–proteomic concordance. AgNPs disrupted oxidative phosphorylation, increased ROS, and altered the expression of mitoribosomal proteins, particularly OPA1, which affected mitochondrial morphology [280]. Using integrated transcriptomic and proteomic analysis and profiling to assess the toxicity of 30 nm AgNPs in silkworm tissue, 43 differentially expressed genes were identified, including TrIa, Pan1, BmGlcNAcase2, Slc46a1, and Tlp, which are involved in digestion, transport, and metabolism. Proteomic validation confirmed consistency with changes at the gene level, especially MIOX and LP-6, indicating oxidative stress and apoptosis [281]. Transcriptomic analysis of Gracilaria edulis-mediated silver nanoparticles (GE-AgNPs, 55–99 nm) as potential agents for cervical cancer therapy revealed downregulation of PI3K, AKT, mTOR and upregulation of PTEN, highlighting the suppression of the PI3K/AKT signalling pathway. GE-AgNPs selectively induced apoptosis in HeLa cells via ROS generation, while sparing normal HEK293 cells. Transcriptomic modulation confirms their cancer-specific molecular effect and therapeutic potential [282]. Transcriptomic profiling of the effects of citrate-coated 20 nm AgNPs (10 μg/mL) on human iPSC-derived hepatocyte-like cells revealed 936 differentially expressed genes, notably massive upregulation of metallothionein (MT1G, MT1M, MT2A) and HSPA6, suggesting oxidative stress. Proteomic data supported these changes, although only 29 genes overlapped, highlighting temporal and mechanistic differences between mRNA and protein responses. The integration of transcriptomics revealed early gene-level perturbations associated with inflammation, metabolism, and cancer pathways, confirming the utility of transcriptomics in assessing AgNP hepatotoxicity [283].

#### 8.1.4. Metabolomics

Metabolomics is the comprehensive analysis of small-molecule metabolites within biological systems, providing a direct snapshot of cellular physiology and metabolic activity. It captures the downstream effects of gene expression and environmental influences, making it a powerful tool for understanding disease mechanisms, identifying biomarkers, and mapping metabolic pathways [284,285,286].

Recent multi-omics research on common carp gills demonstrated that exposure to AgNPs (~11 nm diameter, 0.1 mg/L concentration) led to significant transcriptomic changes, with 687 differentially expressed genes (DEGs), including key metabolic genes such as MDH1, SDHB, ALOX5, and PLA2G4, primarily involved in the TCA cycle and lipid metabolism. These alterations were largely reversible, with only 33 DEGs remaining after a 7-day recovery period [287]. A similar study investigated the metabolomic effects of silver nanoparticles (AgNPs; core size ~12.6 nm, ~93 nm in water) on common carp gills using untargeted LC-MS-based profiling. Although focused on metabolomics, transcriptomic changes are discussed in the context of previous work, which shows that AgNPs alter amino acid and energy metabolism pathways, leading particularly to downregulation of L-histidine, L-isoleucine, L-phenylalanine, and L-glutamate and upregulation of citric acid. These shifts disrupt the TCA cycle and aminoacyl-tRNA biosynthesis [288]. A cross-species transcriptomic meta-analysis identified conserved gene expression changes induced by silver nanoparticles (AgNPs), specifically 20 and 60 nm citrate-coated particles. Transcriptomic data from humans, mice, zebrafish, and C. elegans revealed shared differentially expressed genes associated with oxidative stress, mitochondrial dysfunction, apoptosis, and impaired lipid and amino acid metabolism. Key genes involved in these processes were consistently altered, highlighting mitochondria as a major target of AgNP toxicity [289]. In addition, 30 nm citrate-stabilized silver nanoparticles (AgNPs) were studied on human keratinocytes (HaCaT) using NMR metabolomics. Although transcriptomics was not directly applied, the work links metabolic changes—such as decreased ATP, NAD⁺, and TCA cycle intermediates and increased glutathione (GSH)—to changes in gene expression, particularly in oxidative stress and energy metabolism pathways [290]. Further experiments on these cells investigated the metabolic impact of silver nanoparticles (AgNPs) of different sizes (10, 30, 60 nm) and coatings (citrate, PEG, BSA). The results highlight changes in pathways such as GSH synthesis, glutaminolysis, glycolysis, and the TCA cycle. The metabolic profiles varied according to AgNP size and surface chemistry, highlighting the importance of nanoparticle properties in the cellular response [291]. The cytotoxic effects of tryptone-stabilized silver nanoparticles (T-AgNPs, ~100 μg/mL IC_50_) were studied in MDA-MB-231 mammary tumour cells, highlighting a mechanism involving direct tubulin disruption and impaired microtubule assembly. Proteomic and metabolomic data suggest regulation at the gene level, particularly in lipid metabolism pathways [292]. PEG-coated 30 nm nanoparticles administered intravenously to mice at subtoxic doses (8 mg/kg) caused time- and organ-specific metabolic changes, particularly in the liver and spleen. Glycogen, lipid, and antioxidant metabolism are suggested by changes in transcriptional regulation [293]. The effect of citrate-capped silver nanoparticles (AgNPs, ~23 nm) on gastrointestinal transformation and toxicity using a simulated digestion model and metabolomics in human colonic enterocytes showed that gradually digested AgNPs aggregated to ~100 nm, exhibited increased Ag+ release, and induced ROS generation. Metabolomics revealed disturbances in unsaturated fatty acid biosynthesis and arachidonic acid metabolism, suggesting ferroptosis [294]. The results of these studies indicate the need for comprehensive genomic, transcriptomic, and proteomic analysis.

### 8.2. Organ-on-a-Chip (OoC) Platforms (Microfluidic Models/Microfluidic Chip)

Organ-on-a-chip systems are microfluidic cell culture devices engineered to mimic the physiological functions of human organs. These chips, typically made from transparent polymers like polydimethylsiloxane (PDMS), integrate living human cells into precisely structured environments that replicate tissue–tissue interfaces, fluid flow, and mechanical forces. By simulating aspects of organ-level function, OoCs offer a unique advantage over conventional models, including better prediction of human responses, real-time monitoring, and the ability to create multi-organ interactions [295]. Organ-on-a-chip models have been successfully employed to investigate the toxic effects of AgNPs on various human organs, including the lung, liver, kidney, gut, and skin. Each model allows for the evaluation of organ-specific responses to nanoparticle exposure under dynamic, physiologically relevant conditions [296].

Lung-on-a-Chip: Given that inhalation is a major route of exposure to airborne nanoparticles, lung-on-a-chip devices are pivotal in studying AgNP-induced pulmonary toxicity. These platforms can simulate the air–liquid interface of the alveoli, complete with breathing motions [297]. Studies using lung chips have demonstrated that AgNPs can cause epithelial damage, inflammation, and oxidative stress at realistic exposure levels [298].

Liver-on-a-Chip: The liver plays a central role in metabolizing and detoxifying xenobiotics, including nanoparticles. Liver-on-a-chip systems can maintain functional hepatocytes and microvascular flow, offering insights into AgNP biotransformation, accumulation, and hepatic toxicity [299,300]. This is crucial for understanding dose–response relationships and identifying early biomarkers of liver injury.

Gut-on-a-Chip and Kidney-on-a-Chip: Ingested AgNPs can interact with the gastrointestinal tract before being absorbed and excreted via the kidneys. Gut-on-a-chip models have shown that AgNPs can disrupt epithelial integrity and microbiome balance [301]. Kidney-on-a-chip platforms, on the other hand, help assess the nephrotoxicity and clearance potential of AgNPs under physiologically realistic shear stress and flow [302].

Multi-Organ Chips (Body-on-a-Chip): To understand systemic toxicity, multi-organ chips that link different organ modules (e.g., gut–liver–kidney) have been developed. These integrated systems allow researchers to study how AgNPs are absorbed, distributed, metabolized, and excreted (ADME) and how they elicit multi-organ interactions and toxicities [303].

#### Advantages, Limitations, and Future Perspectives of Organ-on-a-Chip for Nanotoxicology

**Figure 11:**
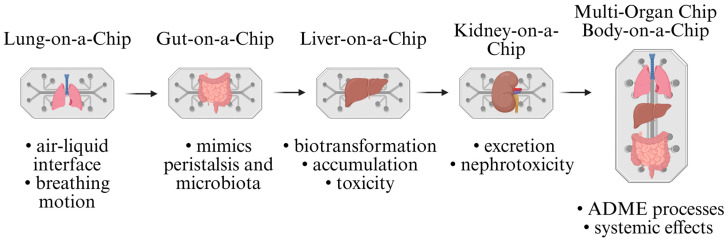
Organ-on-chip model. Original figure made for review using Biorender software.

Organ-on-a-chip technology has physiological relevance because it mimics the dynamic microenvironment of human organs more accurately than static models, and it also offers an ethical alternative to animal testing, thus ensuring a reduction in animal use (Figure 11). Another advantage is real-time monitoring, which allows continuous monitoring of cellular responses and barrier integrity. This model also provides mechanistic insights and facilitates understanding of the molecular pathways involved in nanoparticle-induced toxicity. It is highly modifiable and can be tailored to specific organs, diseases, or patient-derived cells for personalized medicine approaches [304].

While organ-on-a-chip technology represents a significant leap forward, several challenges remain [305]. Standardisation, scalability, and regulatory acceptance are critical hurdles. The material compatibility (e.g., nanoparticle absorption by PDMS), reproducibility, and long-term stability of chips are ongoing technical concerns. Additionally, integrating immune system components and simulating chronic exposure are areas requiring further innovation. In the future, combining OoC platforms with advanced analytical tools like mass spectrometry, transcriptomics, and artificial intelligence can greatly enhance our understanding of nanoparticle safety. Regulatory agencies are beginning to recognize the potential of these models, paving the way for their broader adoption in nanotoxicology and drug development [306].

### 8.3. Radiolabeling Techniques

Radiolabelling involves incorporating radionuclides into nanoparticles, enabling non-invasive visualisation via nuclear imaging techniques such as positron emission tomography (PET), single-photon emission computed tomography (SPECT), and autoradiography [307]. Silver nanoparticles can be radiolabelled either through direct incorporation of radioactive silver isotopes (e.g., ^110m^Ag) or via surface conjugation of chelators bound to isotopes like technetium-99m (^99m^Tc) or iodine-125 (^125^I). Direct radiolabelling with ^125^I has been shown to retain nanoparticle functionality while enabling reliable in vivo tracking [308]. Surface modification with bifunctional chelators such as DTPA or NOTA has also been employed to enhance labelling stability and prevent isotope leaching [309].

Radiolabelled AgNPs have been used to investigate their biodistribution in rodent models. Chrastina et al. (2010) demonstrated predominant accumulation of ^125^I-labeled AgNPs in the liver and spleen following intravenous administration, consistent with uptake by the mononuclear phagocyte system (MPS) [308]. Similar findings have been reported in a study that aimed to investigate the biodistribution and toxicity of radioiodinated Fe_3_O_4_-Ag heterodimer nanoparticles (Fe_3_O_4_-Ag^125^I NPs) after intravenous injection in mice. These nanoparticles are designed for dual-modality imaging using MRI and SPECT. The liver and spleen showed the highest accumulation, consistent with uptake by the mononuclear phagocyte system (Kupffer cells in the liver, macrophages/B cells in the spleen). Some signal in the thyroid and stomach suggested in vivo deiodination (release of free iodine-125). Minimal accumulation was observed in the brain and muscle, suggesting limited systemic distribution outside the RES organs. [310].

The size, shape, and surface chemistry of AgNPs significantly influence their in vivo fate. Smaller nanoparticles exhibit broader tissue penetration and prolonged retention times, potentially increasing their toxicological risk [311]. Surface coatings such as polyethylene glycol (PEG) can prolong circulation time and reduce immune recognition, thereby altering biodistribution patterns [312,313]. Notably, radiolabelling also enables differentiation between intact nanoparticles and their ionic degradation products, addressing a key challenge in nanoparticle toxicology [314]. As silver ions (Ag⁺) are known to contribute to oxidative stress and mitochondrial dysfunction, distinguishing these forms is critical in understanding the mechanisms underlying toxicity [315].

#### Advantages, Limitations, and Future Perspectives of Radiolabelling

Radiolabelling techniques offer several advantages over traditional biodistribution analysis, such as high sensitivity and the ability to detect picomolar to nanomolar concentrations, non-invasive monitoring that allows long-term studies in the same subject, and quantitative imaging allowing real-time measurement of tissue accumulation and clearance. However, there are notable limitations. Isotope selection must account for half-life and imaging resolution needs. Additionally, radiolabel stability is crucial, as isotope dissociation can lead to misleading biodistribution profiles. The synthesis and handling of radiolabelled materials also require specialized facilities and adherence to radiation safety regulations [314]. Radiolabelling will continue to play a central role in the nanotoxicology and pharmacokinetics of AgNPs. Emerging multimodal labelling techniques combining radionuclides with fluorescence or MRI agents offer complementary insights. Furthermore, the development of isotope-specific probes may allow real-time tracking of nanoparticle transformation and ion release in vivo. As regulatory frameworks evolve, radiolabelling data may inform safe-by-design strategies for AgNPs, guiding modifications that reduce off-target accumulation and promote efficient clearance [316,317].

### 8.4. Lipidomics and Interactomics

Lipidomics and interactomics are sophisticated subdivisions of systems biology that are designed to capture the intricacy of cellular responses to external stressors. Lipidomics is the process of the large-scale profiling of cellular lipids, which provides insights into membrane composition, signalling pathways, and energy metabolism. Interactomics, on the other hand, is the study of molecular interactions, particularly those between proteins, nucleic acids, and lipids. These methods are indispensable for comprehending the manner in which toxic agents, including nanoparticles, disrupt cellular homeostasis at both structural and functional levels [318,319]. *Lipidomics* demonstrates the impact of silver nanoparticles (AgNPs) on membrane integrity, lipid metabolism, and oxidative stress responses in the context of AgNP toxicity. Studies have demonstrated that AgNPs, particularly those in the 10–60 nm range, affect the composition of mitochondrial lipids, fatty acid biosynthesis, and pathways such as sphingolipid metabolism and ferroptosis [287,291,294]. AgNP size, surface coating, and ion release are all factors that can prompt cell mortality or inflammation, which are influenced by these disruptions. In a separate recent study, transcriptomic profiling was employed to investigate the impact of metabolic syndrome on the susceptibility to AgNP-induced pulmonary toxicity. The MetS group demonstrated substantially exacerbated responses, as mice exposed to 20 nm citrate-coated AgNPs exhibited elevated expression of inflammatory genes such as IL-6, MIP-2, and MCP-1. Furthermore, AgNP exposure resulted in the downregulation of genes that are involved in lipid metabolism and inflammatory resolution, such as ALOX-5, ALOX-15, and iNOS, with a particular emphasis on MetS mice. These results underscore the significance of evaluating the toxicity of nanoparticles in disease-relevant models, as pre-existing dyslipidaemia and chronic inflammation exacerbate AgNP-induced pulmonary responses [320]. *Interactomic* studies further illustrate how AgNPs disrupt complexes involved in redox balance, apoptosis, and DNA repair by binding to critical residues, thereby interfering with protein–protein and protein–DNA interactions [271,283]. For example, AgNPs contribute to genotoxicity and immunomodulation by impairing mitochondrial protein networks and modulating transcription factors such as p53 and NF-κB [282]. A recent computational study that employed the UnitedAtom interatomic approach assessed the interaction between zero-valent AgNPs and proteins, with a particular emphasis on the formation of protein coronas, which is a critical factor in the biological response. The model simulated the binding affinities of a variety of proteins to AgNPs with varying crystal facets (e.g., (100), (110), (111)). This demonstrated that aromatic and charged amino acids are critical in mediating these interactions and influencing cellular uptake and immune recognition [321]. Furthermore, a bioinformatic and interatomic analysis of proteomic data from Caco-2 cells exposed to AgNPs (11–30 nm) identified key hub proteins—including GAPDH, ENO1, EEF2, and ATP5A1—that were implicated in disrupted protein–protein interaction (PPI) networks. These proteins are essential for the progression of colorectal cancer, as they are involved in pathways related to energy metabolism, translation, and cell adhesion [322]. Combined, lipidomics and interactomics offer a multidimensional perspective on AgNP-induced toxicity, identifying the molecular pathways and biochemical disruptions that are implicated. Their integration provides valuable biomarkers and mechanistic insight for the assessment of the therapeutic and safety potential of nanomaterials.

### 8.5. High-Throughput Screening (HTS)

High-throughput screening (HTS) is a powerful analytical approach that enables the rapid and automated assessment of thousands of compounds or materials for biological activity, originally developed for drug discovery and now widely applied in toxicology and nanomaterial research [323,324]. By integrating robotics, miniaturized assays, and advanced analytics, HTS generates reproducible, high-content data sets while reducing time and cost [325,326]. In nanotoxicology, HTS platforms have been adapted to assess AgNP toxicity under physiologically relevant conditions. A microfluidic-based HTS system revealed greater cytotoxicity of 10 nm AgNPs under dynamic flow compared to static cultures, highlighting the importance of realistic exposure models [327]. Another HTS platform combining CBMN assays with automated cell sorting demonstrated the size- and coating-dependent genotoxicity of PVP-coated AgNPs (10–50 nm), especially in CD2+ and CD4+ lymphocytes, underscoring immune cell-specific vulnerability [328]. These innovations enhance sensitivity, mechanistic insight, and throughput in nanoparticle safety assessment.

## 9. Prediction Models in AgNP Toxicity

### 9.1. Prediction of Dynamic Toxicity of Nanoparticles Using Machine Learning and AI

Nanotoxicology is complicated by the dynamic interactions of silver nanoparticles (AgNPs) with biological systems, influenced by their size, surface chemistry, and environmental transformations [258,329]. Machine learning (ML) offers a predictive alternative to traditional toxicity testing by identifying complex, non-linear relationships between nanoparticle properties and biological outcomes [330,331]. Studies using ML models—including decision trees and random forests—have shown high accuracy in predicting AgNP-induced oxidative stress, cytotoxicity, and genotoxicity, with key predictors including particle size, zeta potential, coating, and exposure time [332,333,334]. These models enable safer-by-design approaches and reduce the reliance on animal studies [335].

### 9.2. Biomarker Identification (e.g., Inflammatory Cytokines)

The identification of specific biomarkers is critical for assessing the biological impact and mechanistic toxicity of silver nanoparticles (AgNPs) [336,337,338,339,340]. Biomarkers commonly associated with AgNP exposure include inflammatory cytokines, oxidative stress markers, and genotoxicity indicators, each reflecting distinct but interconnected pathways of cellular response and injury.

Inflammatory cytokines such as interleukin-1 beta (IL-1β), tumour necrosis factor-alpha (TNF-α), and interleukin-6 (IL-6) are among the most consistently reported biomarkers in AgNP studies. These cytokines signal the activation of immune responses and inflammation. For instance, exposure to polyvinylpyrrolidone (PVP)- and citrate-coated AgNPs (5–50 nm) in human monocytes and macrophages results in a dose- and size-dependent increase in IL-1β, TNF-α, and IL-6 expression, indicating immune cell activation and inflammatory stress [339].

Oxidative stress markers are also prominent in AgNP toxicity profiles. Malondialdehyde (MDA), a lipid peroxidation product, is frequently elevated following AgNP exposure, indicating membrane damage. Concurrently, the activity of antioxidant enzymes such as superoxide dismutase (SOD) and catalase (CAT) often decreases, reflecting impaired redox homeostasis. For example, in RAW264.7 murine macrophages, serum-stabilized AgNPs (~69 nm) were shown to increase intracellular reactive oxygen species (ROS), elevate MDA levels, and reduce SOD activity, collectively demonstrating oxidative stress and redox imbalance [336].

Genotoxicity indicators further elucidate the DNA-damaging potential of AgNPs. Phosphorylation of the histone variant H2AX (γ-H2AX), a marker of DNA double-strand breaks, has been reported in multiple in vitro models exposed to AgNPs. This genotoxic response is often accompanied by apoptotic signalling pathways, such as caspase-3 activation and DNA fragmentation. These responses support the use of γ-H2AX as a sensitive marker for AgNP-induced genomic instability.

In vivo distribution and toxicokinetic studies have also revealed systemic biomarkers associated with organ-specific toxicity. Physiologically based pharmacokinetic (PBPK) modelling and animal models demonstrate that AgNPs accumulate in organs such as the liver, spleen, and kidneys, with associated increases in serum biomarkers, including aspartate aminotransferase (AST), alanine aminotransferase (ALT), and serum creatinine, and hematological parameters like lymphocyte and neutrophil counts [337,340].

Together, these specific biomarkers—spanning inflammation, oxidative stress, genotoxicity, and systemic toxicity—form a robust framework for evaluating the biological effects of AgNPs and guiding safer nanoparticle design and regulatory assessment [338].

### 9.3. Pharmacokinetic (PBPK) Models

Physiologically based pharmacokinetic (PBPK) models are computational instruments that replicate the absorption, distribution, metabolism, and excretion (ADME) of substances, including silver nanoparticles (AgNPs), within biological systems. These models amalgamate physiological information (e.g., organ dimensions, blood circulation) with nanoparticle-specific characteristics (e.g., size, surface coating, dissolution rate) to forecast tissue exposure and potential toxicity. Silver nanoparticles demonstrate toxicity mainly via ion release, oxidative stress, and organ-specific accumulation, influenced by factors like particle size, surface chemistry, and dissolving behaviour [337,341,342]. PBPK models are particularly beneficial in AgNP toxicity investigations, as they provide a mechanistic understanding of biodistribution and bioaccumulation, assist in enhancing risk assessment methodologies, and contribute to diminishing dependence on animal experimentation [343,344]. Complementary modelling methodologies, including QSAR and PBTK models (e.g., the ETH model), offer predictive frameworks that connect physicochemical features to toxicokinetics and organ-specific distribution. The ETH model utilises first-order kinetics and compartmental analysis; nevertheless, it does not provide a mechanistic picture of gastrointestinal changes or the dynamic interactions among silver species. Improvements to traditional QSAR models—integrating non-linear adsorption and thiol-mediated transformations—can more accurately represent rate-limiting stages and saturation kinetics. Mechanistically based models are crucial for regulatory approval and precise human health risk evaluations of AgNPs [345]. Antsiferova et al. illustrated the efficacy of PBPK modelling for nanomaterials by creating a compartmental model that delineates the long-term accumulation kinetics of AgNPs in C57BL/6 mice after chronic oral exposure. Utilising previously published data acquired via neutron activation analysis, their model simulated the distribution of AgNPs and forecasted steady-state concentrations in various organs, including the brain, lungs, liver, and testes, thus addressing concerns regarding systemic toxicity [346].

### 9.4. Quantitative Structure–Activity Relationships (QSAR)

Quantitative Structure–Activity Relationship (QSAR) models assess the toxicity of silver nanoparticles (AgNPs) by linking their physicochemical properties—such as size, shape, surface charge, and coating—with biological effects, including oxidative stress and ion release. These models support safer-by-design strategies and reduce the need for animal testing by enabling efficient, data-driven toxicity prediction [330,347]. Nano-QSAR approaches, including decision tree-based and quasi-QSAR models that incorporate exposure context (e.g., dose, media, duration), have shown strong predictive power for AgNP toxicity [348,349]. Descriptors like surface area, solubility, and LUMO energy have proven critical for classifying toxicological endpoints such as oxidative stress and protein carbonylation [350]. The refinement of nano-specific descriptors remains essential for enhancing model accuracy and regulatory relevance.

## 10. Strategies to Mitigate the Potential Risk Associated with NP-Based Therapies

### 10.1. Surface Modifications

The modification of nanoparticle surfaces is significant as it reduces the toxicity of initial stabilising agents and silver nanoparticles (AgNPs), prevents aggregation, and enhances the targeting capability towards specific cells, particularly cancer cells [351]. According to Gali-Muhtasib et al. [352], an effective anticancer nanocarrier must fulfil several criteria: (i) possess affinity for the anticancer drug to facilitate conjugation; (ii) ensure exclusive drug release at the target site, thereby demonstrating specificity for the tumour; (iii) maintain stability of the nanoparticle–anticancer drug complex in serum; and (iv) degrade in a manner that is safe for the living organism. Currently, the diverse synthesis methods available facilitate the relatively straightforward production of AgNPs, allowing for manipulation to achieve either passive or active targeting [126,353]. Synthesis can involve specific surface ligands to enhance cellular active transport, such as polyethylene glycol (PEG)-poly lactide (PLA), chitosan, silica-based materials, and poly(lactic-co-glycolic acid) (PLGA), or can be tailored to controlled sizes appropriate for passive transport to tumour sites. Passive targeting in tumours is influenced by the presence of defective fenestrated vasculature, characterised by large gaps (ranging from 100 to 800 nm) between endothelial cells and inadequate lymphatic drainage in proximity to tumour sites. The ability of nanoparticles to traverse these vascular gaps is contingent upon their size. Smaller nanoparticles traverse these gaps and accumulate near the tumour, thereby minimising exposure to normal tissues and consequently reducing adverse effects. The enhanced permeability and retention effect (EPR effect) facilitates passive drug delivery to the tumour, providing an additional benefit compared to the free drug. The active targeting of nanoparticles, achieved through the conjugation of a ligand to their surface, demonstrates greater efficiency. This specific ligand can bind selectively to receptors or antigens that are over-expressed on the surface of cancer cells. This method enhances specificity, leading to increased drug uptake and retention at the tumour site, thereby minimizing systemic toxicity [352]. Active targeting represents an enhancement over passive targeting, as it increases the cellular internalisation of nanoparticles by target cells; however, tumour localisation remains unaffected and continues to rely on passive diffusion.

### 10.2. Other Strategies

Among other strategies to reduce the potential harmful effects of AgNPs, coating with biocompatible materials (e.g., polymers or PEG), controlled release mechanisms (e.g., pH or temperature changes), size and shape modification, and comprehensive toxicological evaluation should also be mentioned [354,355].

## 11. Conclusions and Future Perspectives

Silver nanoparticles (AgNPs) have emerged as prospective medicines in oncology, demonstrating strong anticancer capabilities via mechanisms including apoptosis induction, reactive oxygen species generation, and cellular structural disruption. Their distinctive physicochemical properties, like dimensions and surface charge, enable efficient drug administration and focused treatment. Nonetheless, thorough comprehension of their safety profile is still lacking. Potential toxicities, such as oxidative stress, organ-specific damage, and environmental issues, require comprehensive assessment. Recent improvements in green synthesis techniques, employing biological capping agents, provide safer and more environmentally sustainable production alternatives.

So, can we easily answer the question from the title of this article? Do we know enough about safety profile of silver nanoparticles in oncology? The question could be answered from various perspectives. First of all, let us look at the facts we currently do know. AgNPs exhibit promising antineoplastic effects which have been documented in vitro as well as in vivo at preclinical levels. Additionally, AgNPs can synergistically enhance the efficacy of radiation, chemotherapy, and drug delivery methods.

On the other hand, the main areas of uncertainty include: the long-term toxicity of AgNPs, in vivo gaps (although some in vivo animal studies are available, they exhibit considerable variability in approach, and there is a deficiency of substantial human clinical data), and genotoxicity effects (there is a need for more investigation into concerns regarding genotoxicity, pro-inflammatory responses, and oxidative damage in healthy tissues). It is important to mention that, to date, no formulation of silver nanoparticles has received FDA approval for cancer therapy. Their application is confined to preclinical or experimental stages.

In summary, we currently lack sufficient evidence to assert that silver nanoparticles are universally safe in oncology. Significant promise exists; nevertheless, standardized, long-term in vivo and clinical research are essential prior to the widespread adoption of medicinal applications.

Future investigations should prioritize standardized toxicity evaluations, accurate nanoparticle characterisation, and the advancement of surface changes to improve biocompatibility. Confronting these issues is essential for the effective transition of AgNP-based medicines from preclinical research to clinical implementation, guaranteeing both efficacy and safety in cancer treatment.

## Figures and Tables

**Table 1:** Methods of AgNP synthesis.

Bottom-Up Methods			Top-Down Methods
Chemical	Biological		Physical
Reduction	In vivo	In vitro	Milling
Sonochemical	By algae	By biomolecules	Thermal decomposition
Photochemical	By plants	By essential oils	Laser techniques
Microwave assisted	By microorganisms	By cell biomass filtrate	Spray pyrolysis
Electrochemical	By yeasts	Cell-free culture medium	Nanolitography

**Table 2:** Comparison of silver nanoparticles formulations for drug delivery in oncology.

Nanoparticle Formulation	Drug Delivered	Key Features	Citation
Chitosan-coated AgNPs	Tamoxifen	Enhanced cytotoxicity, G2/M cell cycle arrest, apoptosis induction	[129]
Green-synthesized AgNPs	Cisplatin	Synergistic cytotoxicity, reduced IC50 value, increased apoptosis	[130]
Amine-functionalized MSNs-AgNPs	Doxorubicin	High drug loading, uniform shape, small size, biocompatibility	[131]
Carboplatin-loaded AgNPs	Carboplatin	High anticancer activity, low toxicity, pro-apoptotic effects	[132]
Paclitaxel-loaded AgNPs	Paclitaxel	Enhanced anticancer activity, high selectivity, apoptosis induction	[133]
Alginate hydrogel-AgNPs-CisPt	Cisplatin	Synergistic cytotoxic effects, enhanced ROS levels, apoptosis induction	[134]
Hyaluronic acid-QtN-conjugated AgNPs	Quercetin	Targeted delivery, enhanced anticancer efficacy, biocompatibility	[135]
Biosynthesized SeAgNPs	Doxorubicin	High drug encapsulation, biocompatibility, eco-friendly synthesis	[136]
NR1/AgNP-decorated PTX nanocrystals	Paclitaxel	Enhanced cellular uptake, anti-migratory effect, apoptosis induction	[137]
CendR peptide-targeted AgNPs	Monomethyl auristatin E	Selective cytotoxicity, apoptosis induction, targeted delivery	[138]
Silver nanotriangles	Doxorubicin	Synergistic antibreast cancer effect, ROS/ERK1/2 signalling pathway	[139]
PA-AgNPs	Doxorubicin	Targeted delivery, high drug release efficiency, biocompatibility	[140]
CS-AgNPs-DOX-FA	Doxorubicin	Effective drug delivery, apoptosis induction, targeted delivery	[141]

**Table 3:** Principal mechanisms of nano silver toxicity.

Principal Mechanism	A Specific Target/Process Affected by AgNPs Within the Principal Mechanism	References
Oxidative stress	A significant decrease in the content of antioxidant substances ROS production induction; lipid and protein oxidation and DNA damage, resulting in the injury of cellular components and function	[188,189,190]
Endoplasmic reticulum stress	Changes in many ER-stress-related proteins, including phosphorylation of PERK and its downstream eukaryotic initiation factor-2 (eIF-2) and phosphorylated IRE1, endoplasmic-reticulum-stress-specific splicing of x-box transcription factor-1, and cleavage of activated transcription factor 6 (ATF6), splices X-box binding protein 1 (XBP-1 s), and BIP.	[191,192]
Mitochondrial dysfunction via non-ROS pathways	AgNPs penetrate the inner mitochondrial membrane, resulting in the swelling of mitochondria and damage to the ridge structure of mitochondria and influencing mitochondrial fusion and fission As a main consequence, reduced production of energy directly affects the activity of mitochondrial ATP synthase (ATPase), inhibits the respiratory chain, and reduces the production of ATP	[193,194]
Autophagy	abnormalities in autophagosome–lysosome fusion, leading to abnormal accumulation of enlarged autophagosomes in the cytosol with cytotoxic consequences such as DNA damage, mitochondrial impairment, and cell death	[185]
Inflammatory response	Nuclear Factor κB (NF-κb) activation, Interleukins 6–8 (IL-6, IL-8) secretion, Tumour Necrosis Factor-α (TNF-α) and Cyclooxygenase-2 (COX-2) expression by conventional but not green AgNPs	[195]

**Table 4:** In vivo studies evaluating organ toxicity of AgNPs.

Organ	Animal Model	Dose	Exposure Route	End-Point	Toxic Effect	Mechanism	Ref.
Liver	Healthy adult male mice	2 mg·kg^−1^	intraperitoneal injection	35 days	Alterations in the ultrastructure of the liver; focal hepatocytes necrosis and apoptosis	Free radical production and oxidative stress induction	[196]
Lungs	Balb/c mice	0.1 mg/kg body weight	intranasal instillation	1 or 24 h	Impaired lung function	Alterations in lung tissue O_2_ consumption due to increased mitochondrial active respiration and NOX activity leading to oxidative damage.	[197]
Heart	Mice	10^−9^–10^−6 ^g/mL ≥4 mg/kg	intravenous injection	60–90 min	Loss of excitability in mice cardiac papillary muscle cells in vitro associated with sinus bradycardia, complete atrio-ventricular conduction block, and cardiac asystole	Inhibition of the activity of rectifying the inward potassium current (IK1) and inward sodium current (INa) channels of cardiomyocytes, leading to rapid collapse of cardiac cell transmembrane potential (TMP)	[198]
Vaginal mucosa, urethra, and rectum	Healthy female New Zealand rabbits	0.1 g·kg^−1^	intravaginal application	24 and 72 h	Ultrastructural pathological changes to the vaginal mucosa, urethra, and rectum, and the promoted cytotoxic reactions	-	[199]
Fat body and wing imaginal disc	Drosophila melanogaster	50 mg·L^−1^	by ingestion of food	10, 20, and 30 days	Behavioral abnormalities and altered metabolic activity at early larval stage	Impaired essential metabolic Components and increased reactive oxygen species	[200]

**Table 5:** Cellular toxicity of AgNPs detected in vitro.

Cell Line	Toxic Effect(s) and Mechanism(s)	Ref.
**human neural stem cells (NSCs)**	by increasing mitochondrial production of reactive oxygen species led to apoptosis and necrosis of NSCs	[202]
human gingival fibroblast cells	oxidative stress, inflammation, and apoptosis	[203]
normal human lung fibroblast cells (IMR-90),	ROS production or decreased ATP production, resulting in aberrations of the chromosomes and altering energy-dependent DNA repair mechanisms	[204]
HEK-293 cells (human embryonic kidney) cells	direct cytotoxic and viability-lowering effects	[205,206]
human immune cells	IL-1β amount decrease may be related to the impairment of the innate immune response caused by AgNPs toxic effect on the proliferation and expression of human lymphocyte cells and peripheral blood mononuclear cells (PBMCs)	[207,208]

**Table 6:** Classical in vitro assays.

Classical Method	Main Purpose	Main Methods	Principle	Ref.
Size and Surface Charge Evaluation	to understand and predict their biological interactions, toxicity, and environmental impact	dynamic light scattering (DLS) zeta potential (ZP) analysis.	Monitoring of random movements of dilute nanoparticles dispersed in solution caused by Brownian motion (DLS) Identification of the apparent surface charge of nanoparticles (ZP)	[83,209]
Cellular Interaction Assays	to understand nanoparticle’s ability to transport across and interact with cellular barriers	Flow cytometry, confocal microscopy, inductively coupled plasma mass spectroscopy (ICP-MS),TEM, transmission X-ray microscopy (TXM)		[210,211,212]
Viability assays	to assess the toxicity of nanoparticles towards cells	MTT (3-[4,5-dimethylthiazol-2-yl]-2,5 diphenyl tetrazolium bromide), 96AQueous One (96AQ), alamarBlue, LDH, live/dead, neutral red etc.	Enzymatic conversion of dye precursor into a detectable dye within living cells allows evaluating of cell viability and metabolic activity	[213]
Genotoxic assays	to identify potential genotoxic carcinogens and germ cell mutagens	The Ames (the Salmonella/Microsome) test Comet assay in vitro micronucleus (MN) assay	Induction of mutation that reverses a pre-existing mutation in the DNA of a histidine-dependent (his⁻) strain of Salmonella typhimurium (or E. coli), restoring its ability to grow without histidine. Detection of DNA breaks by measuring the migration of fragmented DNA in an electric field (broken DNA fragments migrate away from the nucleus, forming a shape that resembles a comet) Detection of chromosomal damage in cells by identifying the formation of micronuclei—small, extranuclear bodies that contain chromosome fragments or whole chromosomes that were not incorporated into the nucleus after cell division.	[214,215,216,217,218]

**Table 7:** Regulatory in vivo testing.

Toxicity Type	Guideline	Study Durations/Endpoints	Result of Existing AgNP Study:	Ref.
Acute (oral)	e.g., OECD TG 420, 423	Single-dose studies over 14 days.	No deaths or abnormal finding were observed at the maximum concentration for 14 days. LD50 of cAgNPs was considered to be higher than 2000 mg/kg bw in male rats	[220]
Subacute and subchronic	e.g., TG 407, 408	28- or 90-day repeated-dose studies.	Bioaccumulation in liver/spleen at 10 mg/kg	[221]
Carcinogenity and genotoxicity	e.g., TG 451, 471, 474, 487	Long-term studies evaluating tumour risk and genetic damage.	AgNPs (≤ 20 mg/kg) do not cause carcinogenesis in CB6F1 Tg mice via a single-dose intravenous injection	[222]
Reproductive and developmental Toxicity	e.g., TG 414, 421, 422	Assess effects on fertility and offspring development.	The harmful effect of AgNPs on reproductive tissues in female Wistar rats (in a dose of 4 mg/kg of AgNPs)	[223]

## Data Availability

Data are contained within the article.

## Abbreviations

AgNPs	Silver Nanoparticles
AI	Artificial Intelligence
ADME	Absorption, Distribution, Metabolism, and Excretion
ATP	Adenosine Triphosphate
CAM	Chorioallantoic Membrane
DLS	Dynamic Light Scattering
DEG	Differentially Expressed Gene
DNA	Deoxyribonucleic Acid
ER	Endoplasmic Reticulum
FTIR	Fourier Transform Infrared Spectroscopy
HET-CAM	Hen’s Egg Test on the Chorioallantoic Membrane
IL	Interleukin
LDH	Lactate Dehydrogenase
ML	Machine Learning
mRNA	Messenger Ribonucleic Acid
MTT	“3-(4,5-Dimethylthiazol-2-yl)-2,5-Diphenyltetrazolium Bromide”
NPs	Nanoparticles
OoC	Organ-on-a-Chip
PDT	Photodynamic Therapy
PIT	Photoimmunotherapy
PTT	Photothermal Therapy
ROS	Reactive Oxygen Species
RT	Radiotherapy
RT-PCR	Reverse Transcription Polymerase Chain Reaction
SOD	Superoxide Dismutase
TEM	Transmission Electron Microscopy
TNF	Tumour Necrosis Factor
UV/Vis	Ultraviolet Visible Spectroscopy
XPS	X-ray Photoelectron Spectroscopy
XRD	X-ray Diffraction
ZP	Zeta Potential
